# K_2_S_2_O_8_-promoted C–Se bond formation to construct α-phenylseleno carbonyl compounds and α,β-unsaturated carbonyl compounds†

**DOI:** 10.1039/d0ra05927g

**Published:** 2020-08-05

**Authors:** Xue-Yan Yang, Ruizhe Wang, Lu Wang, Jianjun Li, Shuai Mao, San-Qi Zhang, Nanzheng Chen

**Affiliations:** Department of Medicinal Chemistry, School of Pharmacy, Xi'an Jiaotong University Xi'an Shaanxi 710061 PR China smao0115@xjtu.edu.cn; Department of Chemistry, School of Science, MOE Key Laboratory for Nonequilibrium Synthesis and Modulation of Condensed Matter, Xi'an Jiaotong University Xi'an Shaanxi 710049 PR China; The Thoracic Surgery Department of the First Affiliated Hospital of Xi'an Jiaotong University Xi'an Shaanxi 710061 PR China

## Abstract

A novel K_2_S_2_O_8_-promoted C–Se bond formation from cross-coupling under neutral conditions has been developed. A variety of aldehydes and ketones react well using K_2_S_2_O_8_ as the oxidant in the absence of catalyst and afford desired products in moderate to excellent yields. This protocol provides a very simple route for the synthesis of α-phenylseleno carbonyl compounds and α,β-unsaturated carbonyl compounds.

## Abstract 2

K_2_S_2_O_8_-promoted C–Se bond formation from the cross-coupling of C(sp^3^)–H bond adjacent to carbonyl group with diphenyl diselenide under metal-free conditions.

## Graphical abstract

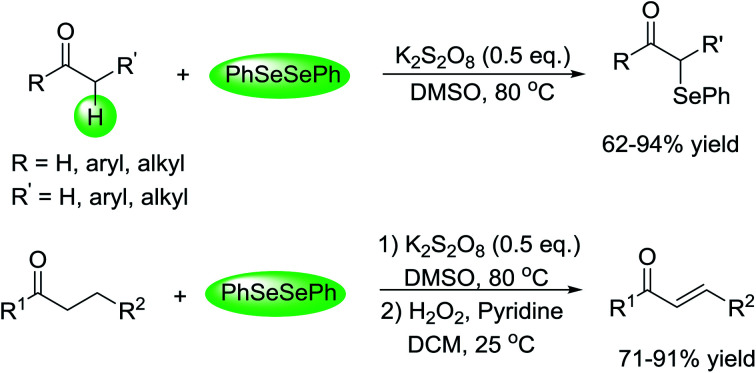

Selenium (Se) is an essential trace mineral nutrient that exerts multiple and complex effects on human health.^1^ Selenium has been widely applied in a variety of fields such as the organic synthesis, catalysis, agriculture chemistry, materials science and even the environment protection.^2^ Se-containing compounds have attracted vast interest because of their extensive bioactive functions and important roles in chemical reactions.^3^ As metabolites of Se in humans, phenylseleno (–SePh) groups are extremely important.^4^ It has been reported that SePh-containing compounds can act as redox agents suitable for targeting cancer cells or play a role in steroid chemistry. Several reported SePh-containing compounds that imitate glutathione peroxidase, like ebselen,^5^ that act as redox agents suitable for targeting cancer cells (naphthoquinone derivatives)^6^ or are important in steroid chemistry (estrogen derivatives)^7^ are shown in Fig. 1. Furthermore, α-phenylseleno carbonyl compounds have a special place since these substances also serve as versatile intermediates in organic synthesis.^8^ They can be converted into the corresponding synthetically useful α,β-unsaturated aldehydes or ketones through oxidation by H_2_O_2_ or NaIO_4_ followed by selenoxide elimination^9^ and Sahani's group has used α-phenylselanyl ketones as substrates to obtain α-arylated ketones through organic photoredox catalysis.^10^

**Fig. 1:**
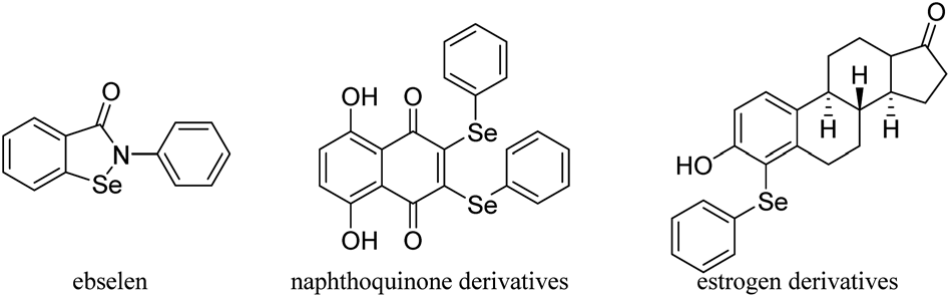
Examples of Se-containing biologically active compounds.

Oxidative functionalization of carbonyl compounds has been known since 1935 (ref. 11) and was studied further by Saegusa, Mislow, Baran and others.^12^ While there generally exist various means, either direct or indirect, of accessing particular target molecules, in order to continue to advance this field, we must constantly study more efficient and green methods. Currently, several procedures have been developed for the preparation of α-phenylseleno aldehydes and ketones. One typical method to synthesize such compounds is by using an enolate coupling reaction.^13^ This approach suffers from the use of a stoichiometric amount of a strong base and metal oxidant to produce the enolate followed by an oxidative coupling reaction (see Scheme 1). In 2015, Yan's group demonstrated that with the participation of a suitable oxidant, ketones can undergo direct oxidation functionalization.^14^ Despite the improvement of not using strong base, it still needed multiple times the amount of metal-free oxidants. In addition, K_2_S_2_O_8_ was found to be a useful oxidant in oxidative reactions because of its characteristics of easy availability, good stability, and low toxicity. Thus, studies focusing on the development of K_2_S_2_O_8_-mediated oxidative reactions meet the requirement of sustainable chemistry.^15^ Based on our research on the functionalization of the C(sp^3^)–H bond, and in connection to our continued interest in developing efficient metal-free functionalization strategies,^16^ herein we report an efficient K_2_S_2_O_8_-mediated C–Se bond formation for the synthesis of α-phenylseleno carbonyl compounds.

**Scheme 1:**
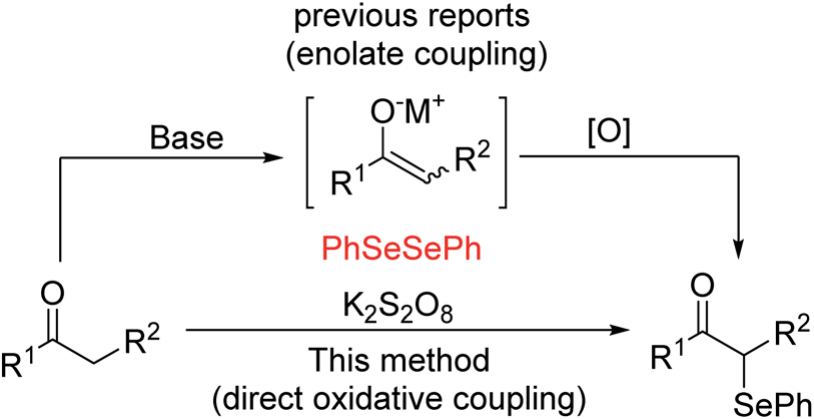
Synthesis of α-phenylseleno carbonyl compounds (M = metal).

Initially, we utilized acetone (1a) as a standard substrate to evaluate the coupling of C(sp^3^)–H bonds adjacent to a carbonyl group with diphenyl diselenide (2). Treatment of 1a with 1.0 equiv. of (NH_4_)_2_S_2_O_8_ in DMSO at 80 °C under air for 3 h afforded the desired product 3a in 29% yield (Table 1, entry 1).^17^ Then various reaction parameters were screened, including the oxidant, solvent, and temperature. A range of oxidants such as PhI(OAc)_2_, IBX, Ag_2_O, Na_2_S_2_O_8_, K_2_S_2_O_8_, and oxone were tested, and K_2_S_2_O_8_ showed the highest efficiency (entries 2–7). The solvent also played a key role in this transformation. The product yield decreased when DMSO was replaced by DMF, DMA, CH_3_CN or EtOH (entries 8–11). Taking the place of air with argon, the reaction gave the desired product 3a in a similar yield (87%) (entry 12). Notably, a similar yield of 3a was obtained by lowering the amount of K_2_S_2_O_8_ to 0.5 equiv. (entry 13). However, a further decrease of the oxidant amount resulted in a lower yield of 3a (entry 14). The reaction temperature had little influence on the reaction efficiency, and 80 °C was still the best choice (entries 15 and 16). A control experiment revealed that K_2_S_2_O_8_ was necessary for the success of this reaction (entry 17).

**Table 1:** Optimization of the reaction conditions^a^^,^^b^

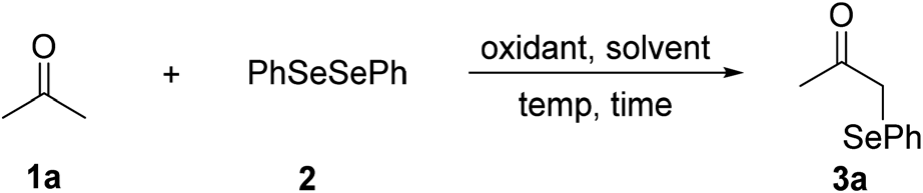				
Entry	Oxidant (equiv.)	Solvent	Temp (°C)/time (h)	Yield^b^ (%)
1	(NH_4_)_2_S_2_O_8_ (1)	DMSO	80/3	29
2	PhI(OAc)_2_ (1)	DMSO	80/6	<5
3	IBX (1)	DMSO	80/6	<5
4	Ag_2_O (1)	DMSO	80/6	n.r.^c^
5	Na_2_S_2_O_8_ (1)	DMSO	80/3	70
6	K_2_S_2_O_8_ (1)	DMSO	80/3	93
7	Oxone	DMSO	80/6	n.r.
8	K_2_S_2_O_8_ (1)	DMF	80/3	52
9	K_2_S_2_O_8_ (1)	DMA	80/3	67
10	K_2_S_2_O_8_ (1)	MeCN	80/3	<5
11	K_2_S_2_O_8_ (1)	EtOH	80/6	<5
12^d^	K_2_S_2_O_8_ (1)	DMSO	80/3	87
**13**	**K**_**2**_ **S**_**2**_ **O**_**8**_ **(0.5)**	**DMSO**	**80/3**	**90 (92)** e
14	K_2_S_2_O_8_ (0.3)	DMSO	80/12	50
15	K_2_S_2_O_8_ (0.5)	DMSO	40/8	85
16	K_2_S_2_O_8_ (0.5)	DMSO	r.t./12^f^	88
17	—	DMSO	80/6	n.r.

a Reaction conditions: 1a (0.5 mmol), 2 (0.25 mmol), oxidant, solvent (2 mL), under air atmosphere.

b Isolated yield based on 1.

c n.r. = no reaction.

d Under argon (1 atm) atmosphere.

e Yield on a 5 mmol scale is given in parentheses.

f Room temperature.

With optimized reaction conditions in hands, we evaluated the scope of the reactions with a variety of ketones. A wide range of acyclic (Table 2, 3a–3c) and cyclic ketones (3d–3j) participated in this process. The mild reaction conditions could tolerate a variety of functional groups, such as aryl (3c). We also observed the yield gradually decreased with the increase of the ketones ring (3d–3h). We then evaluated the scope of the reactions with a variety of aldehydes. Regardless of the length of the side chain (Table 2, 3k–3q), all the aldehydes could smoothly undergo C(sp^3^)–H bond adjacent to carbonyl group selenenylation to afford the corresponding α-phenylseleno carbonyl compounds in good to high yields without changing the standard conditions. Notably, under previous reported conditions, reactions of highly sterically hindered α,α-disubstituted aldehydes occurred in very poor yields, but pleasingly, these substrates were compatible with our reaction system (3s and 3t). Interestingly, condensation adducts were not detected in the course of these reactions when performed under the optimized reaction conditions. Overall, despite the carbon type at α-site of carbonyl, aldehydes and ketones could smoothly undergo C(sp^3^)–H bond selenation to afford the corresponding α-phenylseleno carbonyl compounds in middle to high yields under standard conditions. It's noteworthy that double α-phenylselenenylated adducts were not detected in the course of these reactions.

**Table 2:** Substrates scope for the oxidative coupling^a^^,^^b^

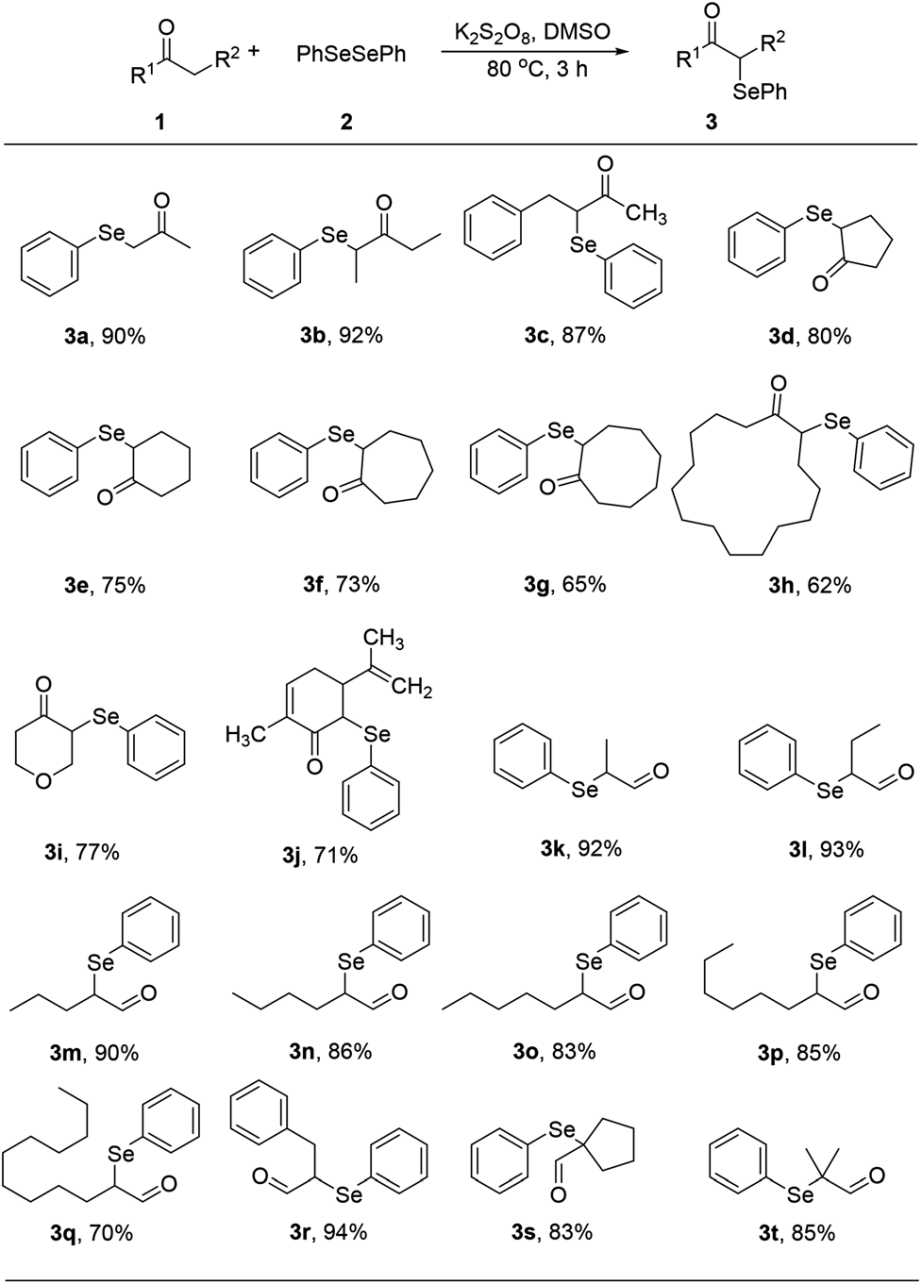

a Reaction conditions: 1 (0.5 mmol), PhSeSePh (0.25 mmol), K_2_S_2_O_8_ (0.25 mmol) and DMSO (2 mL), 80 °C, 3 h.

b Isolated yields based on 1.

As important synthetic intermediates of drug molecules and complex chemicals, α,β-unsaturated carbonyl compounds could be prepared *via* direct α,β-dehydrogenation of ketones and aldehydes using oxidants such 2,3-dichloro-5,6-dicyano-1,4-benzoquinone (DDQ)^18^ and 2-iodoxybenzoic acid (IBX).^19^ Corresponding to direct oxidation methods are stepwise protocols which contain α-substitution of carbonyl compounds and subsequent elimination.

Inspired by the research, and based on above synthesis of α-phenylseleno carbonyl compounds, we attempted to develop an efficient one-pot synthesis of α,β-unsaturated carbonyl compounds from carbonyl compounds. After extensive screening studies, we were pleased to find a desirable protocol; that is, after the reaction of the synthesis of α-phenylseleno carbonyl compounds was complete, H_2_O_2_ and pyridine in dichloromethane were added to the reaction bottle and stirred at 25 °C for 30 min (Table 3). The one-pot approach afforded the desired α,β-unsaturated carbonyl compounds in good yields.

**Table 3:** Conversion of carbonyl compounds to α,β-unsaturated carbonyl compounds^a^

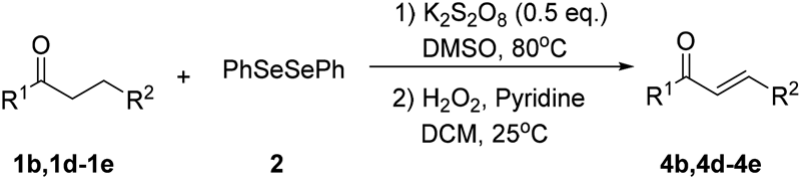			
Entry	Substrates	Products	Yield
1	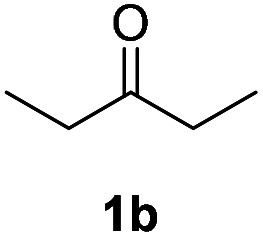	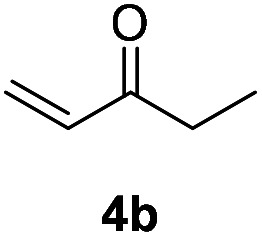	71%
2	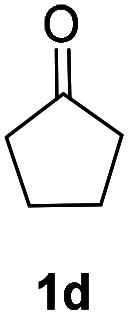	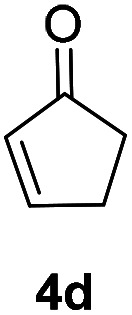	91%
3	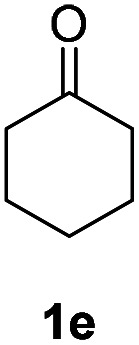	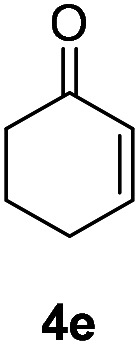	87%

a All reactions were performed with 1 (0.5 mmol), PhSeSePh (0.25 mmol), K_2_S_2_O_8_ (0.25 mmol) and DMSO (2 mL) at 80 °C under air for 3 h. After the UV absorption of diphenyl diselenyl ether completely disappeared (monitored by TLC), the mixture was cooled to 25 °C, and then H_2_O_2_ (1.5 mmol), pyridine (1 mmol), DCM (3 mL) were added. The mixture was stirred at 25 °C for 30 min. Isolated yields based on 1.

We next focused on the mechanism of this reaction. First, two radical-trapping reagents, 2,2,6,6-tetramethylpiperidin-1-oxyl (TEMPO) and 1,1-diphenylethylene (DPE) were added to the standard reaction system respectively. It was found that no product 3a was detected in both reactions (Scheme 2a and b). Then a visible light-promoted experiment was investigated. The reaction of 1a with 2 irradiated by a 18 W white LED in DMSO gave 3a in 41% yield after 12 hours (Scheme 2c). These results shown that the reaction may involve a radical process.

**Scheme 2:**
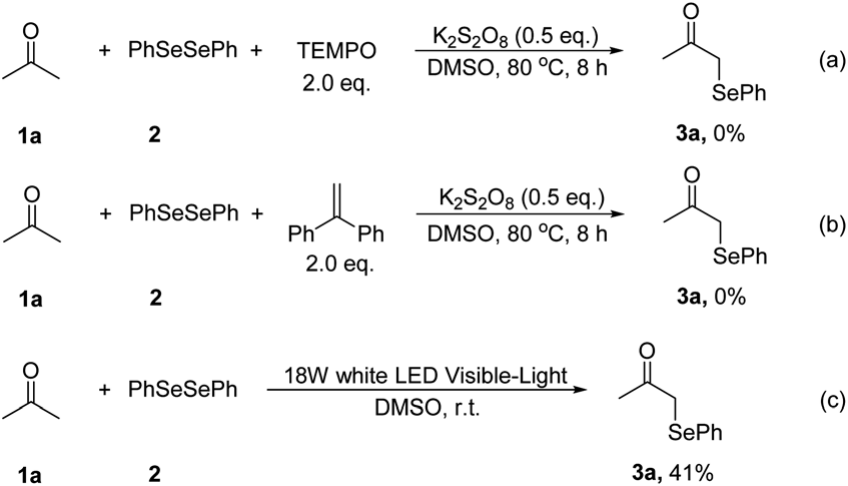
Control experiments for mechanistic study.

Based on the experimental results and related reports, a possible mechanism was proposed (Scheme 3). In the initiation stage, S_2_O_8_^2−^ decomposed to form sulfate radicals SO_4_˙^−^ by breaking of the O–O bond under heat condition.^20^ Then SO_4_˙^−^ reacted with carbonyl compounds to generate racial A. In the chain propagation stage, radical A reacted with diphenyl diselenide 2 to produce the α-phenylseleno carbonyl compounds product 3 and a phenylseleno radical B, which then reacted with another molecule of A to generate a new α-phenylseleno carbonyl compounds. Under oxidative conditions, 3 was oxidized to give α-carbonyl selenoxides C, which could decompose into α,β-unsaturated carbonyl compounds 4*via* selenoxide elimination.

**Scheme 3:**
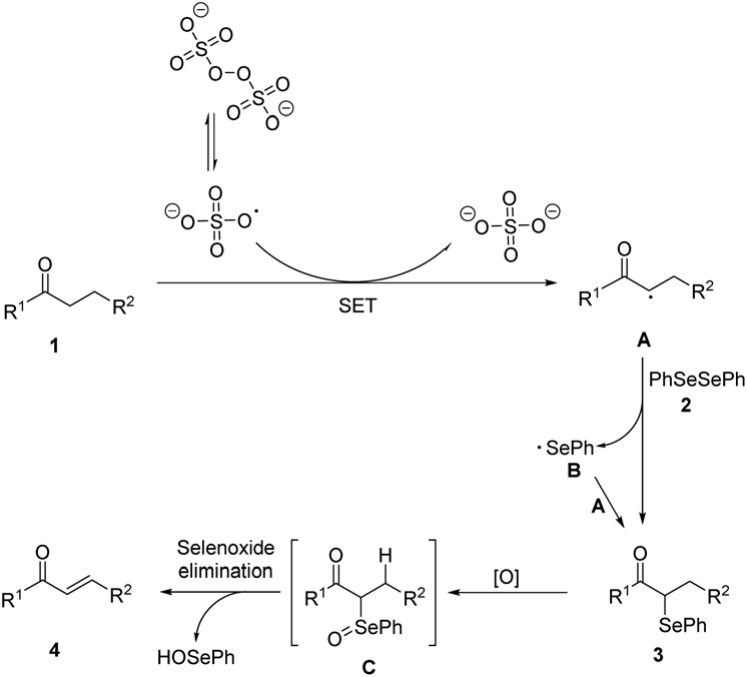
Proposed reaction mechanism.

## Conclusions

In conclusion, we have developed a novel K_2_S_2_O_8_-mediated oxidative coupling of C(sp^3^)–H bonds adjacent to carbonyl group with diselenides, which provided a direct and efficient entry to the α-phenylseleno carbonyl compounds in moderate to excellent yields. The couplings were followed by oxidation elimination to efficiently synthesize α,β-unsaturated carbonyl compounds. The two protocols feature economic process, wide substrate scope and high functional-group tolerance. Tentative mechanistic studies suggest that the coupling reaction is likely to proceed by a single-electron transfer (SET). Further studies of the scope and limitations of this green approach to the synthesis of selenium-containing compounds of biological and pharmaceutical interest are currently under investigation.

## Conflicts of interest

There are no conflicts to declare.

## Supplementary Material

RA-010-D0RA05927G-s001
