# Supplementary material for: K2S2O8-promoted C–Se bond formation to construct α-phenylseleno carbonyl compounds and α,β-unsaturated carbonyl compounds
Source: RSC Adv. 2020 Aug 5;10(48):28902–5. doi: 10.1039/d0ra05927g (PMC9055883; doi:10.1039/d0ra05927g)
Supplement: RA-010-D0RA05927G-s001 [file RA-010-D0RA05927G-s001.pdf]

Electronic Supplementary Material (ESI) for RSC Advances.
This journal is © The Royal Society of Chemistry 2020

Supporting Information

$K_2S_2O_8$ –Promoted C–Se Bond Formation to Construct *α*-Phenylseleno Carbonyl Compounds and *α,β*-Unsaturated Carbonyl Compounds

Xue-Yan Yang,‡[a] Rui-Zhe Wang,‡[b] Lu Wang,[a] Jian-Jun Li,[b] Shuai Mao,*[a] San-Qi Zhang*[a], Nanzheng Chen*[c]

[a]*Department of Medicinal Chemistry, School of Pharmacy, Xi'an Jiaotong University, Xi'an, Shaanxi, 710061, PR China*
[b]*Department of Chemistry, School of Science, and MOE Key Laboratory for Nonequilibrium Synthesis and Modulation of Condensed Matter, Xi'an Jiaotong University, Xi'an, Shaanxi, 710049, PR China.*
[c]*The thoracic surgery department of the First affiliated hospital of Xi'an Jiaotong University, Xi'an, Shaanxi, 710061, PR China.*

Table of Contents

General information..S-2

Experimental Information and Characterization Data..S-2

Reference..S-10

NMR spectra...S-11

General information

All the regular chemicals were purchased from commercial sources with purity over 95% and used without further purification. ^{1}H and ^{13}C NMR spectra were recorded on a Bruker 400 MHz NMR (400 MHz and 100 MHz, respectively) spectrometer or a Bruker 600 MHz NMR (600 MHz and 150 MHz, respectively) spectrometer. Chemical shifts (δ) was expressed in ppm. Flash chromatography was performed using standard grade silica gel (200-300 mesh). Analytical TLC was performed with GF254 precoated silica gel plates with visualization using UV (254 nm) radiation. High resolution mass spectra (HRMS) were acquired with a Bruker Daltonics MicroTof-Q II mass spectrometer. Products purification was done using silica gel column chromatography. Infrared (IR) data were recorded as films on potassium bromide plates with a Bruker Tensor 27 FT-IR spectrometer. Absorbance frequencies are reported in reciprocal centimeters (cm^{-1}).

Experimental Information and Characterization Data

General procedure for the synthesis of 3a-3t.

Carbonyl compounds (0.5 mmol), PhSeSePh (0.25 mmol), and $K_2S_2O_8$ (0.25 mmol) were placed in a 10 mL sealed tube. Dimethyl sulfoxide (2 mL) was added, and the mixture was heated at 80℃. TLC was used to monitor the progress of the reaction until the UV absorption of diphenyl diselenide completely disappeared or the UV absorption of the target product was no longer enhanced. After the reaction was completed, the mixture was cooled to room temperature and then transferred to a separating funnel with ethyl acetate (80 mL), washed with water (20 mL×10), saturated brine (30 mL×2), and the organic phase was dried over anhydrous sodium sulfate and filtered. The filtrate was evaporated by rotary evaporation to remove the organic solvent, and the residue was separated by column chromatography to obtain the pure target product.

General procedure for the synthesis of 4b, 4d-4e.

Carbonyl compounds (0.5 mmol), PhSeSePh (0.25 mmol), and $K_2S_2O_8$ (0.25 mmol) were placed in a 10 mL sealed tube. Dimethyl sulfoxide (2 mL) was added, and the

mixture was heated at 80℃. When the UV absorption of PhSeSePh disappeared by TLC monitoring, the mixture was cooled to 25℃, and then H_2O_2 (1.5 mmol, 150 μL 30% H_2O_2), pyridine (1 mmol), DCM (3 mL) were added. The mixture was stirred for additional 30 min then added to 25 ml of dichloromethane and 30 ml of 7% $NaHCO_3$ solution. The aqueous layer was washed with 25 ml of dichloromethane, and the combined organic layers were washed with 30 ml of 10% HCl solution and 30 ml of saturated NaCl and dried (Na_2SO_4). After solvent removal, distillation gave the target product.

Control experiments for mechanistic study.

Reaction of Scheme 2a-b: **1a** (0.5 mmol), PhSeSePh (0.25 mmol), $K_2S_2O_8$ (0.25 mmol), TEMPO (1 mmol) or DPE (1 mmol) were placed in a 10 mL sealed tube. Dimethyl sulfoxide (2 mL) was added, and the mixture was heated at 80℃ for 8 h. TLC was used to monitor the progress. Reaction of Scheme 2c: **1a** (0.5 mmol) and PhSeSePh (0.25 mmol) were placed in a 10 mL sealed tube. Dimethyl sulfoxide (2 mL) was added, and the mixture was stired at room temperature for 12 h under the light from a 18W white LED. The post-processing was the same as that of **3a-3t**.

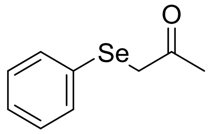

1-(Phenylselanyl)propan-2-one.[1] Compound **3a** was obtained in 90% yield (96.3 mg) according to the general procedure (PE/EA, 75:1~50:1): yellow oil; ^{1}H NMR (400 MHz, $CDCl_3$) δ 7.56 – 7.49 (m, 2H), 7.32 – 7.26 (m, 3H), 3.59 (s, 2H), 2.27 (s, 3H); ^{13}C NMR (100 MHz, $CDCl_3$) δ 203.6, 133.3, 129.4, 128.8, 128.0, 36.9, 28.1; HRMS (ESI) calcd for $C_9H_{11}OSe$: $[M+H]^+$ 214.9975, found: 214.9977.

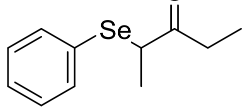

2-(Phenylselanyl)pentan-3-one.[2] Compound **3b** was obtained in 92% yield (111.4 mg) according to the general procedure (PE/EA, 75:1): yellow oil; ^{1}H NMR (400 MHz, $CDCl_3$) δ 7.54 – 7.49 (m, 2H), 7.36 – 7.27 (m, 3H), 3.81 (q, *J* = 7.0 Hz, 1H),

2.85 – 2.73 (m, 1H), 2.55 – 2.43 (m, 1H), 1.48 (d, *J* = 7.0 Hz, 3H), 1.08 (t, *J* = 7.3 Hz, 3H); ^{13}C NMR (100 MHz, $CDCl_3$) δ 207.7, 135.9, 129.2, 128.8, 127.2, 45.1, 33.1, 16.6, 8.5; HRMS (ESI) calcd for $C_{11}H_{15}OSe$: $[M+H]^+$ 243.0288, found: 243.0289.

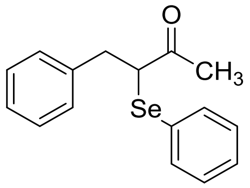

4-phenyl-3-(phenylselanyl)butan-2-one.[1] Compound **3c** was obtained in 87% yield (132.1 mg) according to the general procedure (PE/EA, 50:1): yellow liquid; ^{1}H NMR (400 MHz, $CDCl_3$) δ 7.48 (d, 2H), 7.35 (t, 1H), 7.29 (dd, 4H), 7.21 (t, 1H), 7.16 (d, 2H), 3.92 (dd, 1H), 3.24 (dd, 1H), 3.01 (dd, 1H), 2.24 (s, 3H); ^{13}C NMR (100 MHz, $CDCl_3$) δ 203.6, 139.1, 135.8, 129.3, 129.0, 128.9, 128.5, 127.3 ,126.6, 53.0, 36.8, 28.3; HRMS (ESI) calcd for $C_{16}H_{17}OSe$: $[M+H]^+$ 305.0445, found: 305.0447.

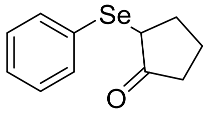

2-(Phenylselanyl)cyclopentan-1-one.[1] Compound **3d** was obtained in 80% yield (96.4 mg) according to the general procedure (PE/EA, 60:1): yellow oil; ^{1}H NMR (400 MHz, $CDCl_3$) δ 7.63 – 7.56 (m, 2H), 7.34 – 7.28 (m, 3H), 3.79 – 3.71 (m, 1H), 2.38 – 2.26 (m, 2H), 2.24 – 2.12 (m, 1H), 2.10 – 1.87 (m, 3H); ^{13}C NMR (100 MHz, $CDCl_3$) δ 214.6, 135.4, 129.2, 128.5, 127.9, 46.5, 36.4, 30.8, 21.0; HRMS (ESI) calcd for $C_{11}H_{13}OSe$: $[M+H]^+$ 241.0132, found: 241.0135.

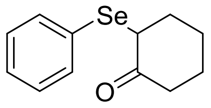

2-(Phenylselanyl)cyclohexan-1-one.[3] Compound **3e** was obtained in 75% yield (95.2 mg) according to the general procedure (PE/EA, 75:1): yellow oil; ^{1}H NMR (400 MHz, $CDCl_3$) δ 7.56 – 7.52 (m, 2H), 7.29 (m, 3H), 3.94 – 3.89 (m, 1H), 3.03 – 2.93 (m, 1H), 2.36 – 2.26 (m, 1H), 2.24 – 2.15 (m, 2H), 1.97 (m, 1H), 1.88 – 1.67 (m, 3H); ^{13}C NMR (100 MHz, $CDCl_3$) δ 207.9, 134.6, 129.2, 128.6, 128.1, 51.6, 38.5, 34.0, 26.9, 22.9; HRMS (ESI) calcd for $C_{12}H_{15}OSe$: $[M+H]^+$ 255.0288, found: 255.0290.

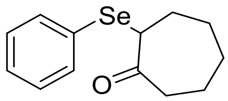

2-(Phenylselanyl)cycloheptan-1-one.[4] Compound **3f** was obtained in 73% yield (97.5 mg) according to the general procedure (PE/EA, 50:1): yellow oil; ^{1}H NMR (400 MHz, $CDCl_3$) δ 7.55 – 7.53 (m, 2H), 7.28 – 7.24 (m, 3H), 3.79 (dd, *J* = 11.1, 5.6 Hz, 1H), 2.71 (dd, *J* = 16.9, 7.3 Hz, 1H), 2.42 – 2.32 (m, 1H), 2.31 – 2.20 (m, 1H), 1.88 (dd, *J* = 14.1, 7.4 Hz, 2H), 1.84 – 1.76 (m, 1H), 1.67 – 1.55 (m, 1H), 1.51 – 1.33 (m, 2H), 1.24 (q, *J* = 11.3 Hz, 1H); ^{13}C NMR (100 MHz, $CDCl_3$) δ 209.2, 134.9, 129.1, 128.5, 128.3, 52.4, 40.0, 30.6, 30.3, 28.1, 25.7; HRMS (ESI) calcd for $C_{13}H_{17}OSe$: $[M+H]^+$ 269.0445, found: 269.0446.

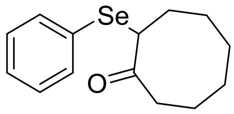

2-(Phenylselanyl)cyclooctan-1-one.[5] Compound **3g** was obtained in 65% yield (91.6 mg) according to the general procedure (PE/EA, 50:1): yellow oil; ^{1}H NMR (400 MHz, $CDCl_3$) δ 7.55 – 7.50 (m, 2H), 7.34 – 7.26 (m, 3H), 3.74 – 3.69 (m, 1H), 2.83 (td, *J* = 12.4, 3.6 Hz, 1H), 2.25 (ddd, *J* = 12.4, 5.2, 3.6 Hz, 1H), 2.15 – 2.05 (m, 2H), 1.86 (ddd, *J* = 11.5, 8.9, 4.6 Hz, 1H), 1.80 – 1.51 (m, 5H), 1.33 – 1.22 (m, 1H), 1.15 (m, 1H); ^{13}C NMR (100 MHz, $CDCl_3$) δ 211.0, 135.3, 129.2, 128.5, 128.0, 53.0, 36.8, 29.2, 28.7, 28.4, 25.7, 24.2; HRMS (ESI) calcd for $C_{14}H_{19}OSe$: $[M+H]^+$ 283.0601, found: 283.0604.

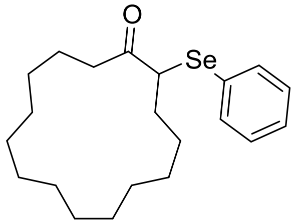

2-(Phenylselanyl)cyclopentadecan-1-one.[6] Compound **3h** was obtained in 62% yield (117.8 mg) according to the general procedure (PE/EA, 75:1): yellow semi-solid; ^{1}H NMR (400 MHz, $CDCl_3$) δ 7.53 – 7.49 (m, 2H), 7.34 – 7.27 (m, 3H), 3.75 (dd, *J* = 9.9, 5.1 Hz, 1H), 2.69 – 2.60 (m, 1H), 2.54 – 2.42 (m, 1H), 2.01 – 1.93 (m, 1H), 1.75 – 1.64 (m, 2H), 1.57 – 1.43 (m, 1H), 1.27 (s, 20H); ^{13}C NMR (100 MHz, $CDCl_3$) δ

207.6, 135.6, 129.1, 128.6, 127.5, 50.9, 40.0, 30.5, 27.5, 27.4, 27.0, 26.9, 26.8, 26.7, 26.3, 26.2, 26.1, 26.1, 24.3; HRMS (ESI) calcd for $C_{21}H_{33}OSe$: $[M+H]^+$ 381.1697, found: 381.1699.

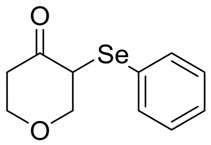

3-(phenylselanyl)tetrahydro-4H-pyran-4-one.[4] Compound **3i** was obtained in 77% yield (98.6 mg) according to the general procedure (PE/EA, 70:1): yellow liquid; ^{1}H NMR (400 MHz, CDCl3) δ 7.59 – 7.53 (m, 2H), 7.30 (d, 3H), 4.08 (d, 3H), 3.98 – 3.87 (m, 2H), 3.17 – 3.07 (m, 1H), 2.54 – 2.43 (m, 1H); ^{13}C NMR (100 MHz, CDCl3) δ 203.1, 134.8, 129.3, 128.4, 127.6, 73.0, 68.3, 51.1, 40.3; HRMS (ESI) calcd for $C_{11}H_{13}O_2Se$: $[M+H]^+$ 257.0081, found: 257.0084.

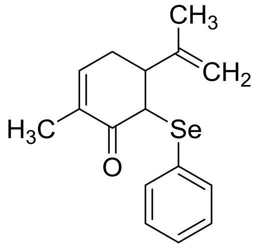

2-methyl-6-(phenylselanyl)-5-(prop-1-en-2-yl)cyclohex-2-en-1-one. Compound **3j** was obtained in 71% yield (108.6 mg) according to the general procedure (PE/EA, 50:1): yellow liquid; ^{1}H NMR (600 MHz, $CDCl_3$) δ 7.57 – 7.52 (m, 2H), 7.37 – 7.28 (m, 2H), 7.25 (d, J = 7.1 Hz, 1H), 6.67 (d, J = 5.6 Hz, 1H), 5.04 (d, J = 1.0 Hz, 1H), 4.90 (s, 1H), 4.06 (d, J = 3.1 Hz, 1H), 2.90 (d, J = 10.9 Hz, 1H), 2.69 – 2.59 (m, 1H), 2.40 – 2.29 (m, 1H), 1.79 (s, 3H), 1.76 (d, J = 1.0 Hz, 3H); ^{13}C NMR (150 MHz, $CDCl_3$) δ 195.4, 143.8, 143.0, 136.2, 133.7, 129.0, 128.4, 127.0, 112.4, 51.6, 45.0, 27.7, 21.7, 16.2; HRMS (ESI) calcd for $C_{16}H_{19}OSe$: $[M+H]^+$ 307.0601, found: 307.0603. IR (cm^{-1}): 3055, 2920, 2850, 1664, 1577, 1477, 1436, 1367, 1096, 1055, 1022.

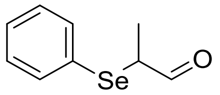

2-(Phenylselanyl)propanal.[3] Compound **3k** was obtained in 92% yield (98.4 mg)

according to the general procedure (PE/EA, 70:1): yellow oil; ^{1}H NMR (400 MHz, $CDCl_3$) δ 9.45 (d, *J* = 2.7 Hz, 1H), 7.55 – 7.49 (m, 2H), 7.37 – 7.28 (m, 3H), 3.72 (dd, *J* = 7.0, 2.7 Hz, 1H), 1.46 (d, *J* = 7.0 Hz, 3H); ^{13}C NMR (100 MHz, $CDCl_3$) δ 193.6, 136.2, 129.3, 129.0, 125.7, 46.6, 13.5; HRMS (ESI) calcd for $C_9H_{11}OSe$: $[M+H]^+$ 214.9975, found: 214.9978.

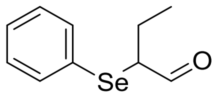

2-(Phenylselanyl)butanal.[3] Compound **3l** was obtained in 93% yield (106.0 mg) according to the general procedure (PE/EA, 75:1): yellow oil; ^{1}H NMR (400 MHz, $CDCl_3$) δ 9.41 (d, J = 3.5 Hz, 1H), 7.53 – 7.48 (m, 2H), 7.36 – 7.27 (m, 3H), 3.53 (td, J = 7.4, 3.5 Hz, 1H), 1.92 – 1.83 (m, 1H), 1.73 – 1.66 (m, 1H), 1.07 (t, J=7.4Hz, 3H); ^{13}C NMR (100 MHz, $CDCl_3$) δ 193.1 136.0, 129.3, 128.9, 125.9, 54.8, 21.1, 12.7; HRMS (ESI) calcd for $C_{10}H_{13}OSe$: $[M+H]^+$ 229.0132, found: 229.0135.

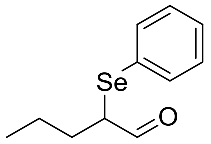

2-(Phenylselanyl)pentanal.[3] Compound **3m** was obtained in 90% yield (108.9 mg) according to the general procedure (PE/EA, 75:1): yellow oil; ^{1}H NMR (400 MHz, $CDCl_3$) δ 9.38 (d, *J* = 3.7 Hz, 1H), 7.51 – 7.49 (m, 2H), 7.41 – 7.27 (m, 3H), 3.64 – 3.57 (m, 1H), 1.85 – 1.75 (m, 1H), 1.71 – 1.62 (m, 1H), 1.60 – 1.41 (m, 2H), 0.94 (t, *J* = 7.3 Hz, 3H); ^{13}C NMR (100 MHz, $CDCl_3$) δ 193.1, 135.9, 129.3, 128.9, 126.0, 52.7, 29.7, 21.3, 13.8; HRMS (ESI) calcd for $C_{11}H_{15}OSe$: $[M+H]^+$ 243.0288, found: 243.0290.

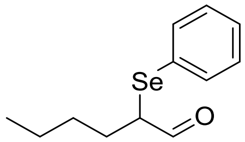

2-(Phenylselanyl)hexanal.[4] Compound **3n** was obtained in 86% yield (110.1 mg) according to the general procedure (PE/EA, 60:1): yellow oil; ^{1}H NMR (400 MHz, $CDCl_3$) δ 9.38 (d, *J* = 3.7 Hz, 1H), 7.59 – 7.48 (m, 2H), 7.39 – 7.27 (m, 3H), 3.65 – 3.55 (m, 1H), 1.91 – 1.77 (m, 1H), 1.75 – 1.62 (m, 1H), 1.55 – 1.46 (m, 2H), 1.42 –

1.33 (m, 2H), 0.90 (t, *J*= 7.1 Hz, 3H); ^{13}C NMR (100 MHz, $CDCl_3$) δ 193.1, 135.9, 129.3, 128.9, 126.0, 53.0, 30.1, 27.3, 22.4, 13.9; HRMS (ESI) calcd for $C_{12}H_{17}OSe$: $[M+H]^+$ 257.0445, found: 257.0448.

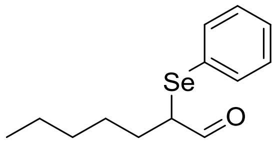

2-(Phenylselanyl)heptanal.[4] Compound **3o** was obtained in 83% yield (112.1 mg) according to the general procedure (PE/EA, 70:1): yellow oil; ^{1}H NMR (400 MHz, $CDCl_3$) δ 9.38 (d, J = 3.7 Hz, 1H), 7.51 (d, 2H), 7.31 – 7.27 (m, 3H), 3.60 (td, J = 7.4, 3.7 Hz, 1H), 1.88 – 1.76 (m, 1H), 1.72 – 1.60 (m, 1H), 1.58 – 1.48 (m, 1H), 1.45 – 1.36 (m, 1H), 1.36 – 1.23 (m, 4H), 0.89 (t, J = 6.5 Hz, 3H). ^{13}C NMR (100 MHz, $CDCl_3$) δ 193.1, 135.8, 129.3, 128.8, 126.0, 53.0, 31.4, 27.7, 27.6, 22.4, 14.0; HRMS (ESI) calcd for $C_{13}H_{19}OSe$: $[M+H]^+$ 271.0601, found: 271.0603.

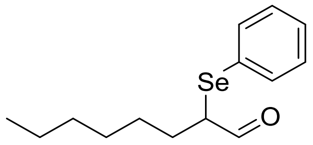

2-(Phenylselanyl)octanal.[4] Compound **3p** was obtained in 85% yield (120.7 mg) according to the general procedure (PE/EA, 70:1): yellow oil; ^{1}H NMR (400 MHz, $CDCl_3$) δ 9.37 (d, J = 3.7 Hz, 1H), 7.58 – 7.48 (m, 2H), 7.40 – 7.27 (m, 3H), 3.59 (td, J = 7.4, 3.7 Hz, 1H), 1.91 – 1.76 (m, 1H), 1.73 – 1.60 (m, 1H), 1.54 – 1.22 (m, 8H), 0.87 (t, J = 6.6 Hz, 3H); ^{13}C NMR (100 MHz, $CDCl_3$) δ 193.1, 135.9, 129.3, 128.8, 126.0, 53.0, 31.6, 28.9, 28.0, 27.8, 22.6, 14.1; HRMS (ESI) calcd for $C_{14}H_{21}OSe$: $[M+H]^+$ 285.0758, found: 285.0761.

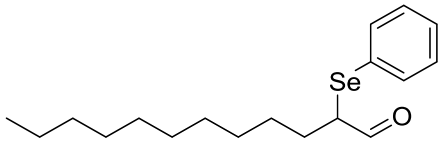

2-(Phenylselanyl)dodecanal.[4] Compound **3q** was obtained in 70% yield (119.0 mg) according to the general procedure (PE/EA, 75:1~40:1): yellow oil; ^{1}H NMR (400 MHz, $CDCl_3$) δ 9.37 (d, *J* = 3.7 Hz, 1H), 7.53 – 7.48 (m, 2H), 7.35 – 7.25 (m, 3H), 3.59 (td, *J* = 7.4, 3.7 Hz, 1H), 1.90 – 1.73 (m, 1H), 1.72 – 1.61 (m, 2H), 1.26 (s, 15H),

0.88 (t, J = 6.7 Hz, 3H); ^{13}C NMR (100 MHz, $CDCl_3$) δ 193.1, 135.9, 129.3, 128.8, 126.0, 53.0, 31.9, 29.6, 29.5, 29.4, 29.3, 28.3, 28.0, 27.7, 22.7, 14.2; HRMS (ESI) calcd for $C_{18}H_{29}OSe$: $[M+H]^+$ 341.1384, found: 341.1386.

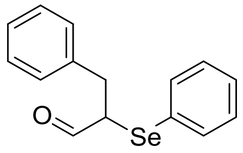

3-Phenyl-2-(phenylselanyl)propanal.[4] Compound **3r** was obtained in 94% yield (136.3 mg) according to the general procedure (PE/EA, 70:1): yellow oil; ^{1}H NMR (400 MHz, $CDCl_3$) δ 9.45 (d, *J* = 3.0 Hz, 1H), 7.51 – 7.45 (m, 2H), 7.36 – 7.31 (m, 1H), 7.31 – 7.22 (m, 5H), 7.20 – 7.17 (m, 2H), 3.87 (ddd, *J* = 8.4, 6.6, 3.0 Hz, 1H), 3.22 (dd, *J* = 14.5, 8.4Hz, 1H), 2.98 (dd, *J* = 14.5, 6.6 Hz, 1H); ^{13}C NMR (100 MHz, $CDCl_3$) δ 192.1, 138.3, 136.1, 129.4, 129.1, 129.1, 128.7, 126.9, 125.8, 53.5, 34.1; HRMS (ESI) calcd for $C_{15}H_{15}OSe$: $[M+H]^+$ 291.0288, found: 291.0291.

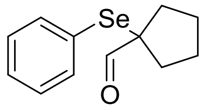

1-(Phenylselanyl)cyclopentane-1-carbaldehyde.[7] Compound **3s** was obtained in 83% yield (105.4 mg) according to the general procedure (PE/EA, 60:1): yellow oil; ^{1}H NMR (400 MHz, $CDCl_3$) δ9.36 (s, 1H), 7.52 – 7.47 (m, 2H), 7.41 – 7.32 (m, 1H), 7.30 – 7.27 (m, 2H), 2.15 – 2.08(m, 2H), 1.88 – 1.83(m, 2H), 1.80 – 1.73 (m, 2H), 1.62 – 1.58(m, 2H); ^{13}C NMR (100 MHz, $CDCl_3$) δ 193.3, 136.8, 129.3, 129.1, 127.0, 63.1, 32.2, 24.7; HRMS (ESI) calcd for $C_{12}H_{15}OSe$: $[M+H]^+$ 255.0288, found: 255.0291.

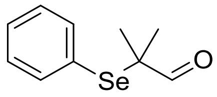

2-Methyl-2-(phenylselanyl)propanal.[4] Compound **3t** was obtained in 85% yield (96.8 mg) according to the general procedure (PE/EA, 75:1): yellow oil; ^{1}H NMR (400 MHz, $CDCl_3$) δ 9.28 (s, 1H), 7.53 – 7.50 (m, 2H), 7.43 – 7.38 (m, 1H), 7.34 – 7.29 (m, 2H), 1.46 (s, 6H); ^{13}C NMR (100 MHz, $CDCl_3$) δ 193.5, 137.8, 129.6, 129.1, 126.1, 53.4, 21.5; HRMS (ESI) calcd for $C_{10}H_{13}OSe$: $[M+H]^+$ 229.0132, found: 229.0136.

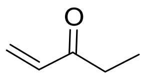

Pent-1-en-3-one.[8] Compound **4b** was obtained in 71% yield (29.8 mg) according to the general procedure (PE/EA, 70:1): Colorless liquid; ^{1}H NMR (400 MHz, $CDCl_3$) δ 6.33 (dd, *J* = 17.7, 10.5 Hz, 1H), 6.23 – 6.14 (m, 1H), 5.79 (dd, *J* = 10.5, 0.9 Hz, 1H), 2.59 (q, *J* = 7.3 Hz, 2H), 1.08 (t, *J* = 7.3 Hz, 3H); ^{13}C NMR (100 MHz, $CDCl_3$) δ 201.4, 136.4, 127.8, 32.8, 7.9; HRMS (ESI) calcd for C_5H_9O: $[M+H]^+$ 85.0653, found: 85.0656.

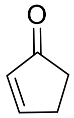

Cyclopent-2-en-1-one.[9] Compound **4d** was obtained in 91% yield (37.3 mg) according to the general procedure (PE/EA, 70:1): Amber liquid; ^{1}H NMR (400 MHz, $CDCl_3$) δ 7.74 (dt, *J* = 5.4, 2.6 Hz, 1H), 6.22 (dt, *J* = 5.6, 2.2 Hz, 1H), 2.71 (dq, *J* = 6.9, 2.3 Hz, 2H), 2.42 – 2.31 (m, 2H); ^{13}C NMR (100 MHz, $CDCl_3$) δ 210.7, 165.0, 134.5, 34.0, 29.0; HRMS (ESI) calcd for C_5H_7O: $[M+H]^+$ 83.0497, found: 83.0499.

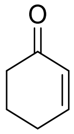

Cyclohex-2-en-1-one.[9] Compound **4e** was obtained in 87% yield (41.7 mg) according to the general procedure (PE/EA, 80:1): yellow liquid; ^{1}H NMR (400 MHz, $CDCl_3$) δ 7.01 (dt, *J* = 10.1, 4.1 Hz, 1H), 6.02 (dt, *J* = 10.1, 2.0 Hz, 1H), 2.47 – 2.40 (m, 2H), 2.36 (tdd, *J* = 6.1, 4.1, 2.0 Hz, 2H), 2.03 (dt, *J* = 12.3, 6.1 Hz, 2H); ^{13}C NMR (100 MHz, $CDCl_3$) δ 199.3, 150.7, 129.8, 38.1, 25.6, 22.7; HRMS (ESI) calcd for C_6H_9O: $[M+H]^+$ 97.0653, found: 97.0656.

Reference

1 Y. Cao, J. Liu, F. Liu, L. Jiang and W. Yi, *Org. Chem. Front.*, 2019, **6**, 825.

2 M. Nazari and B. Movassagh, *Tetrahedron Lett.*, 2009, **50**, 1453.

3 F. N. Victoria, C. S. Radatz, M. Sachini, R. G. Jacob, G. Perin, W. P. da Silva and E. J. Lenardão, *Tetrahedron Lett.*, 2009, **50**, 6761.

4 J. Wang, H. Li, Y. Mei, B. Lou, D. Xu, D. Xie, H. Guo and W. Wang, *J. Org. Chem.*, 2005, **70**, 5678.

5 C. Janine and F. Nathalie, *Tetrahedron Lett.*, 1993, **34**, 7755.

6 L. Engman, *J. Org. Chem.*, 1988, **53**, 4031.

7 L. Set, D. R. Cheshire and D. L. J. Clive, *J. Chem. Soc. Chem. Commun.*, 1985, **1985**, 1205.
8 T. Veysoglu, L. A. Mitscher and J. K. Swayze, *Synthesis*, 1980, **1980**, 807.
9 H. Tateno, Y. Miseki and K. Sayama, *Chem. Commu.*, 2019, **55**, 9339.

NMR spectra

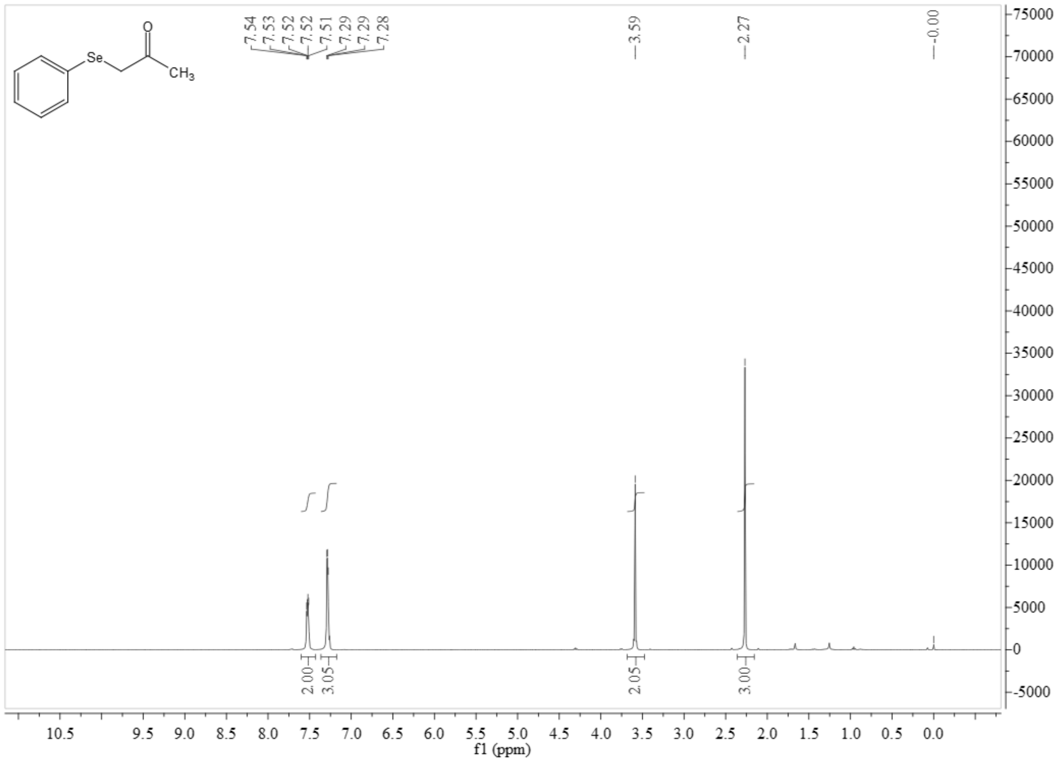

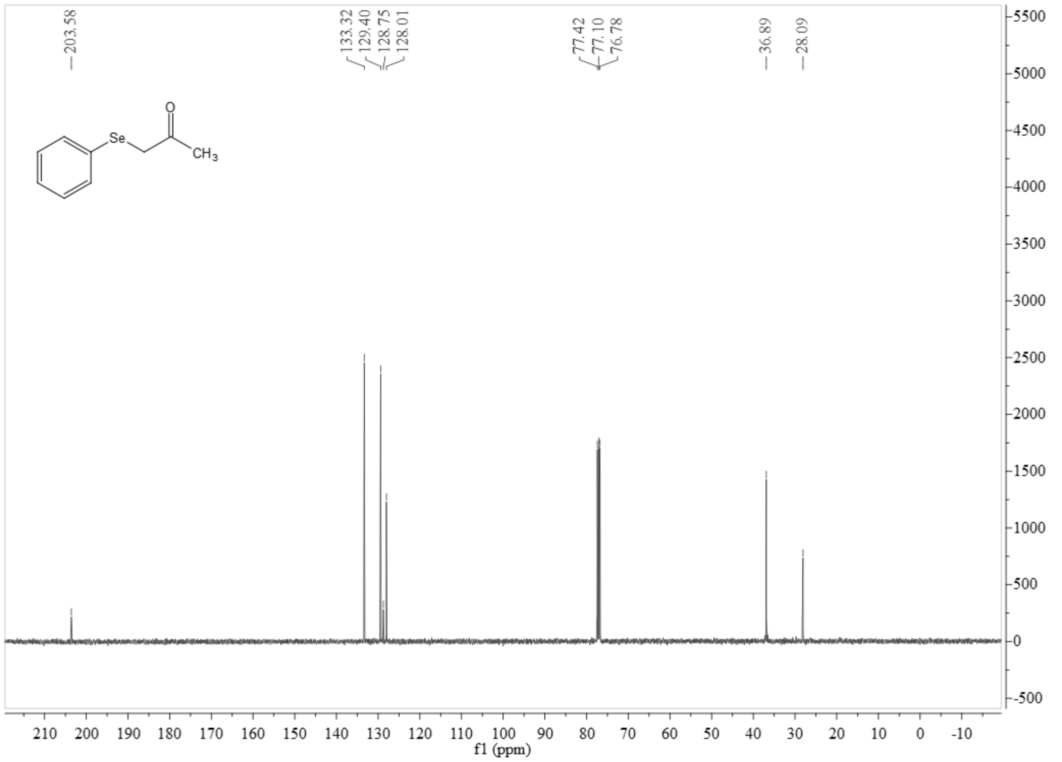

Figure S1. ^{1}H NMR and ^{13}C NMR of **3a**

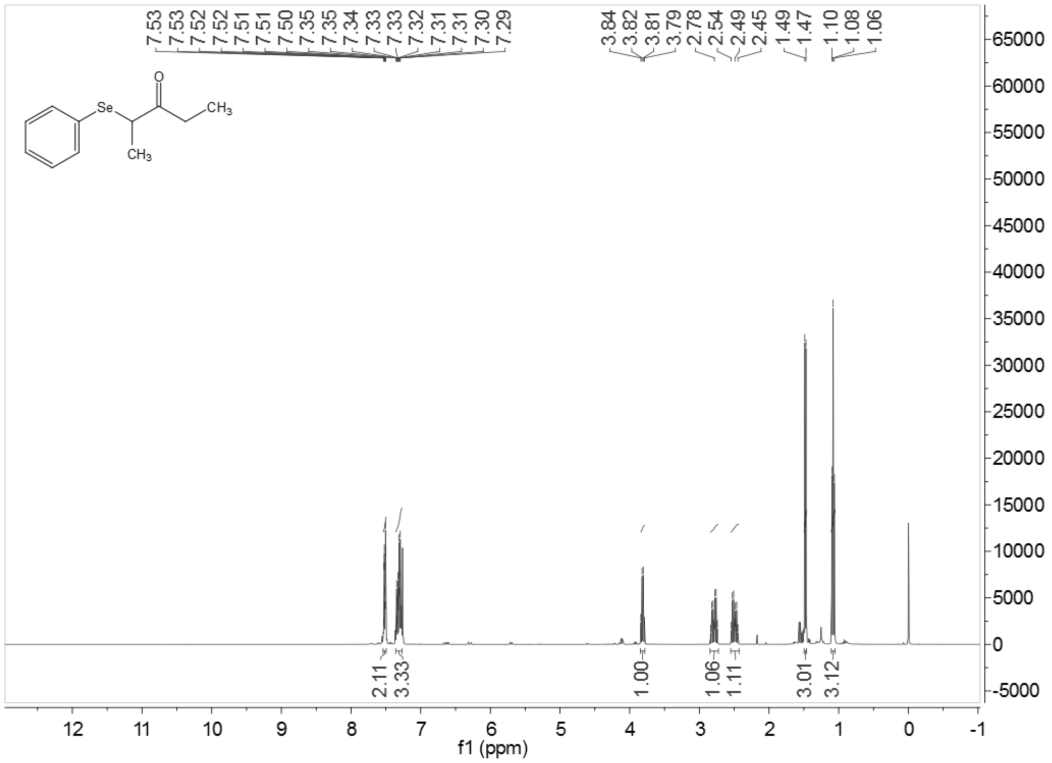

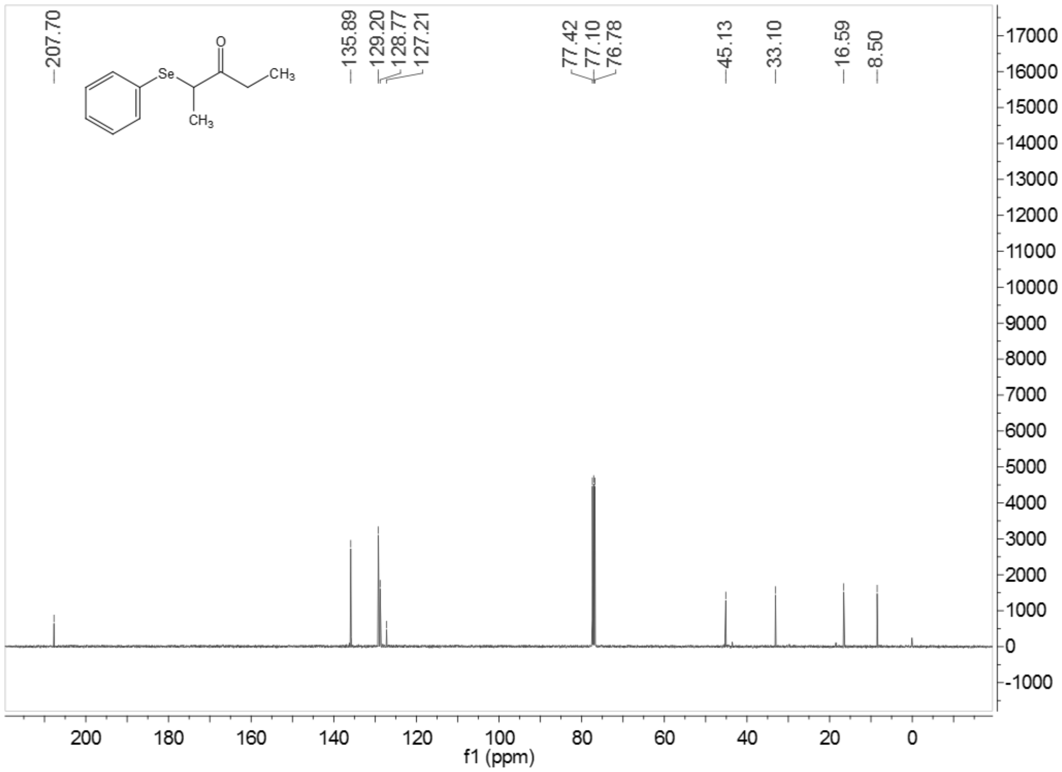

Figure S2. ^{1}H NMR and ^{13}C NMR of **3b**

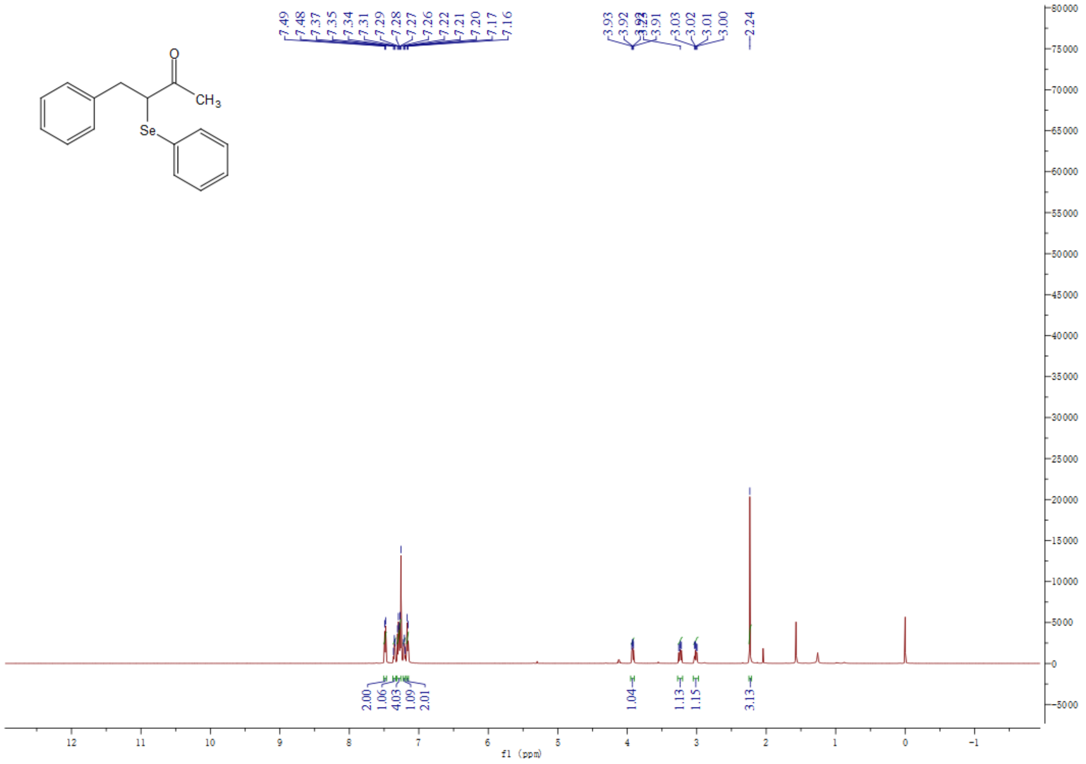

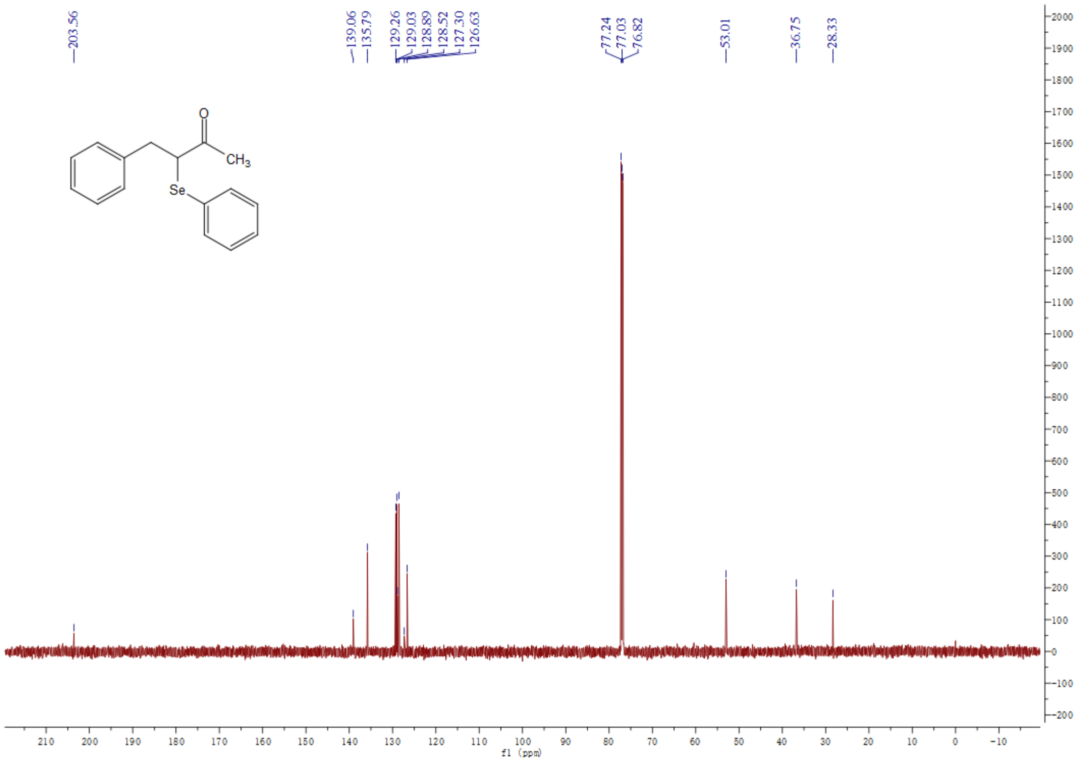

Figure S3. ^{1}H NMR and ^{13}C NMR of **3c**

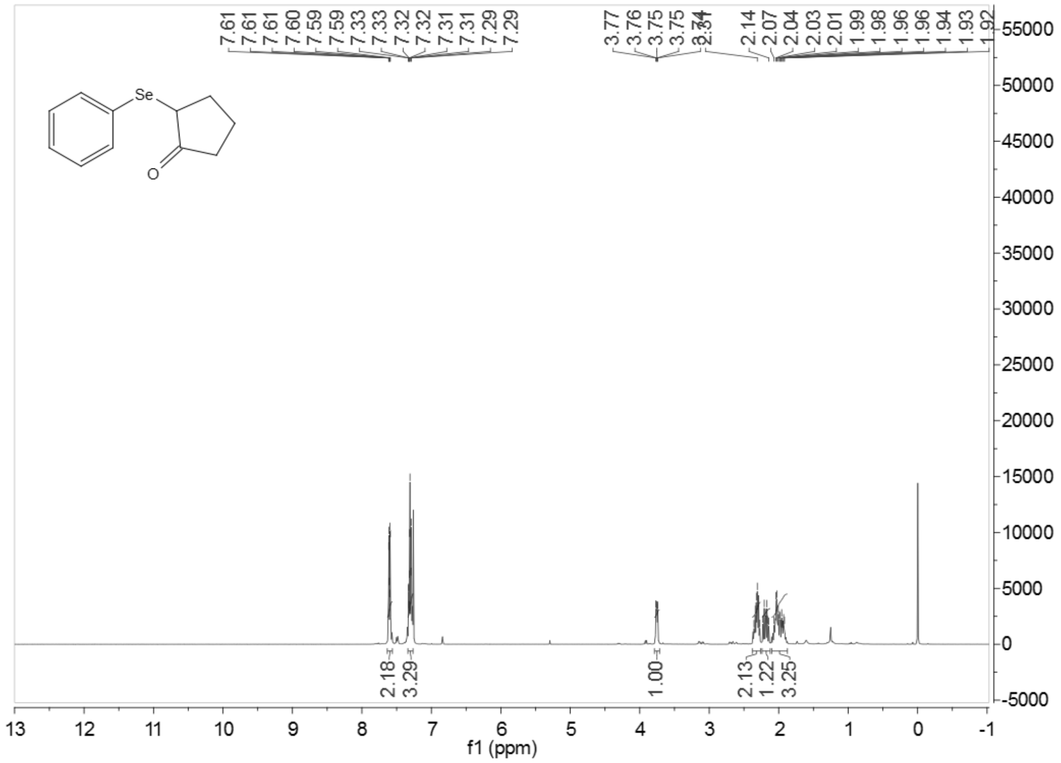

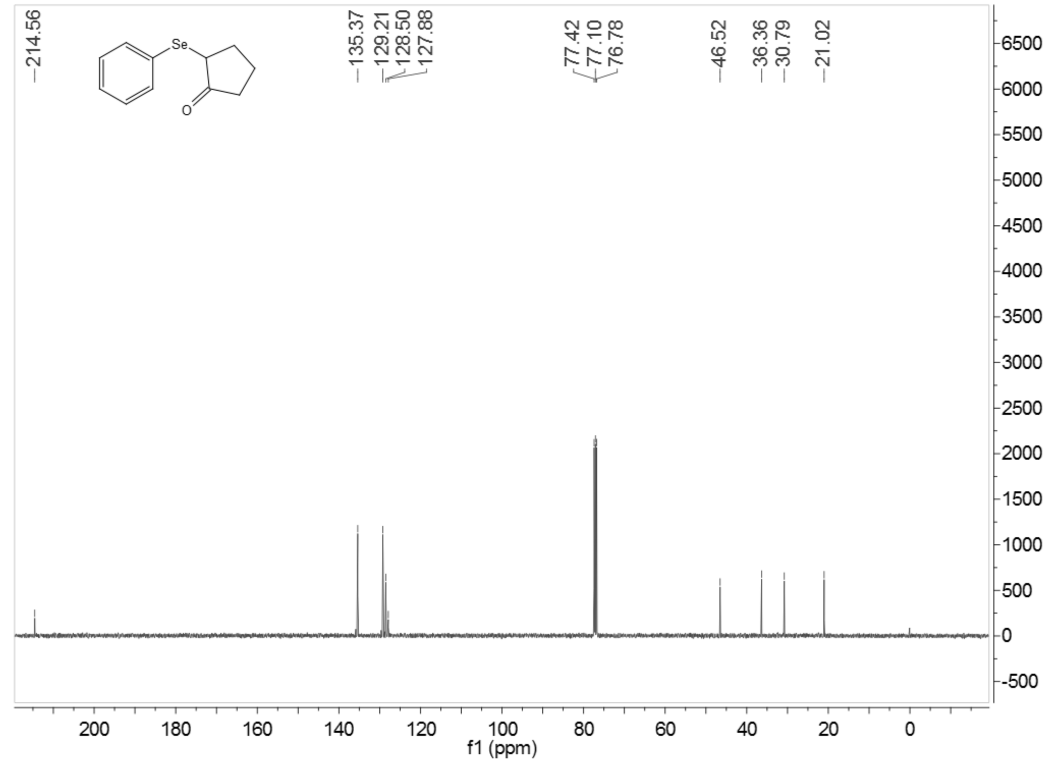

Figure S4. ^{1}H NMR and ^{13}C NMR of **3d**

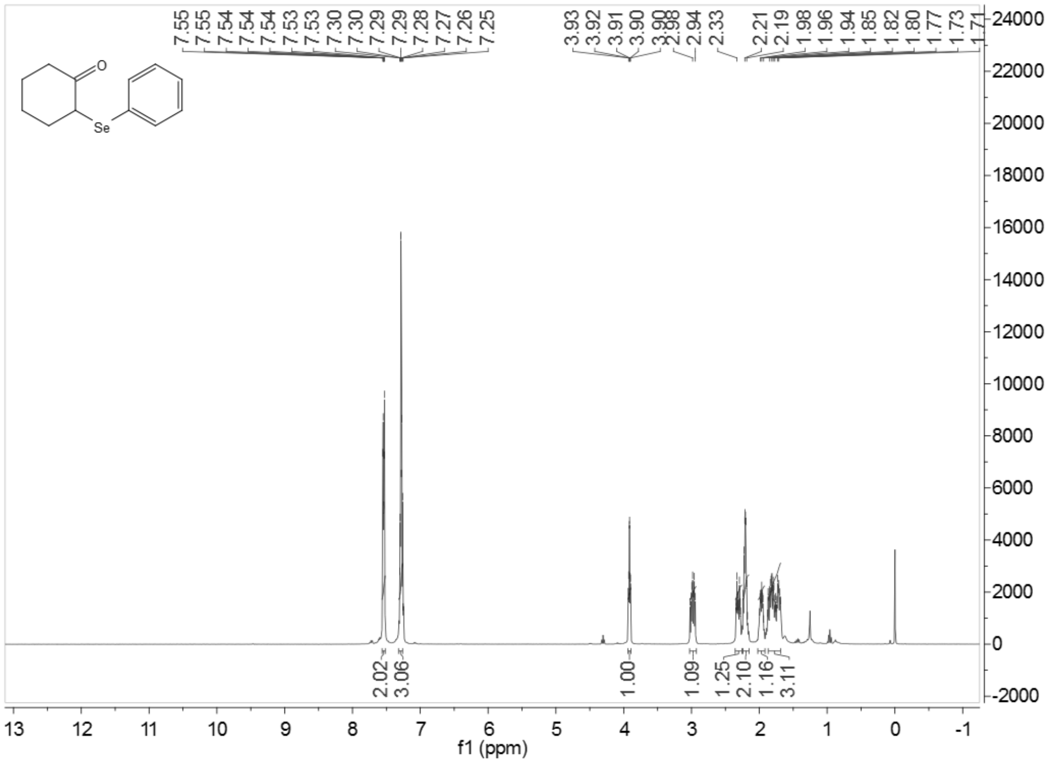

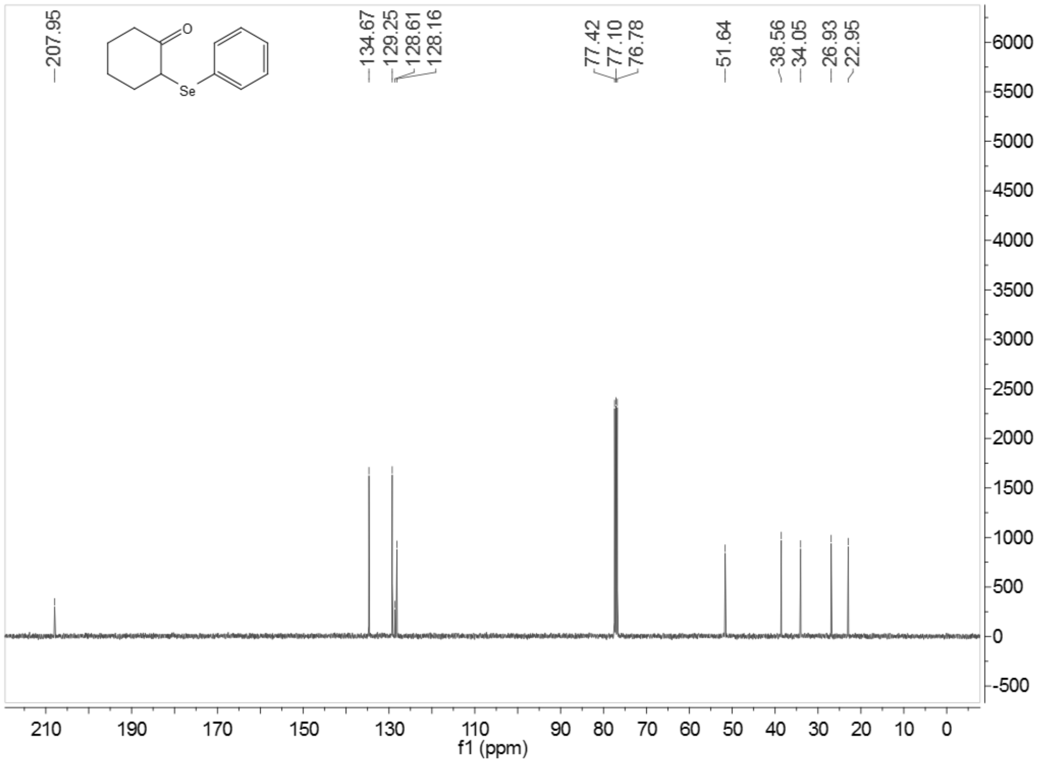

Figure S5. ^{1}H NMR and ^{13}C NMR of **3e**

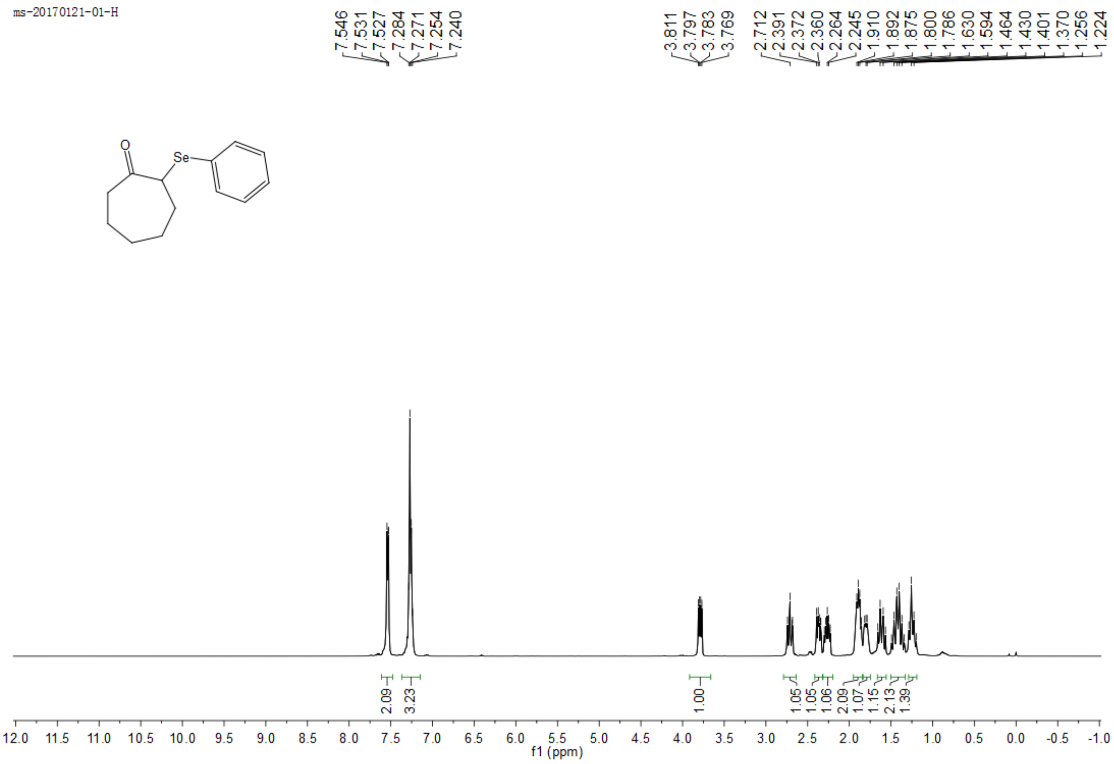

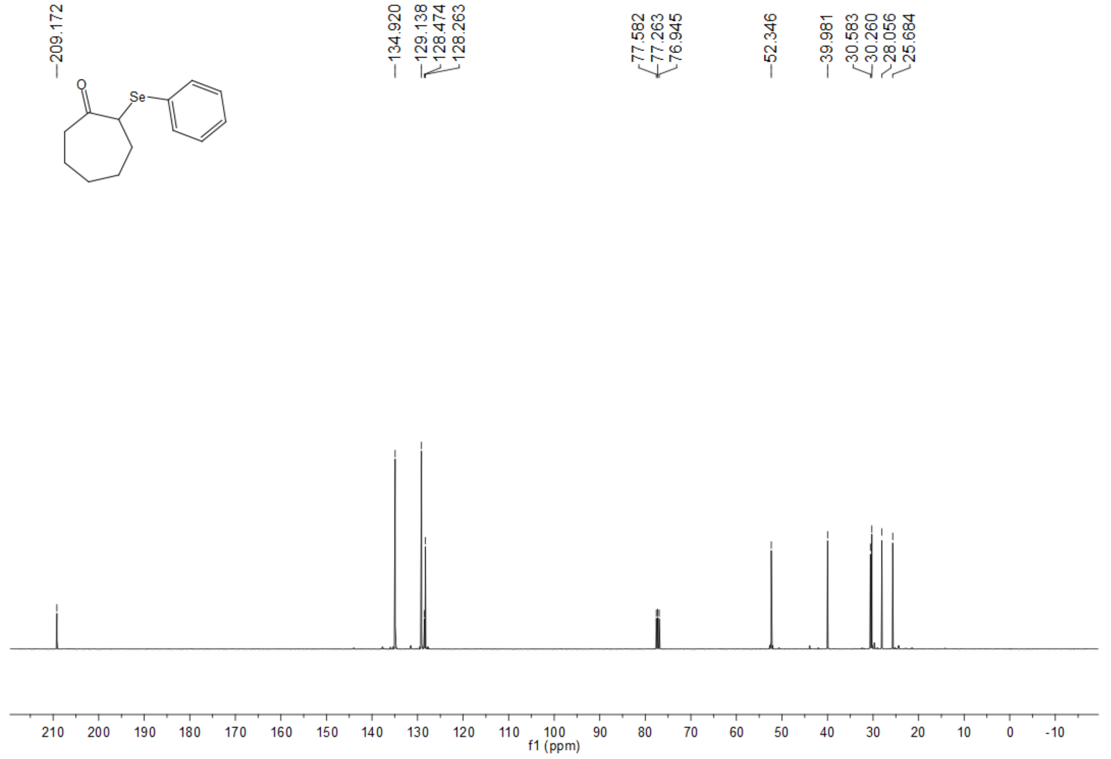

Figure S6. ^{1}H NMR and ^{13}C NMR of **3f**

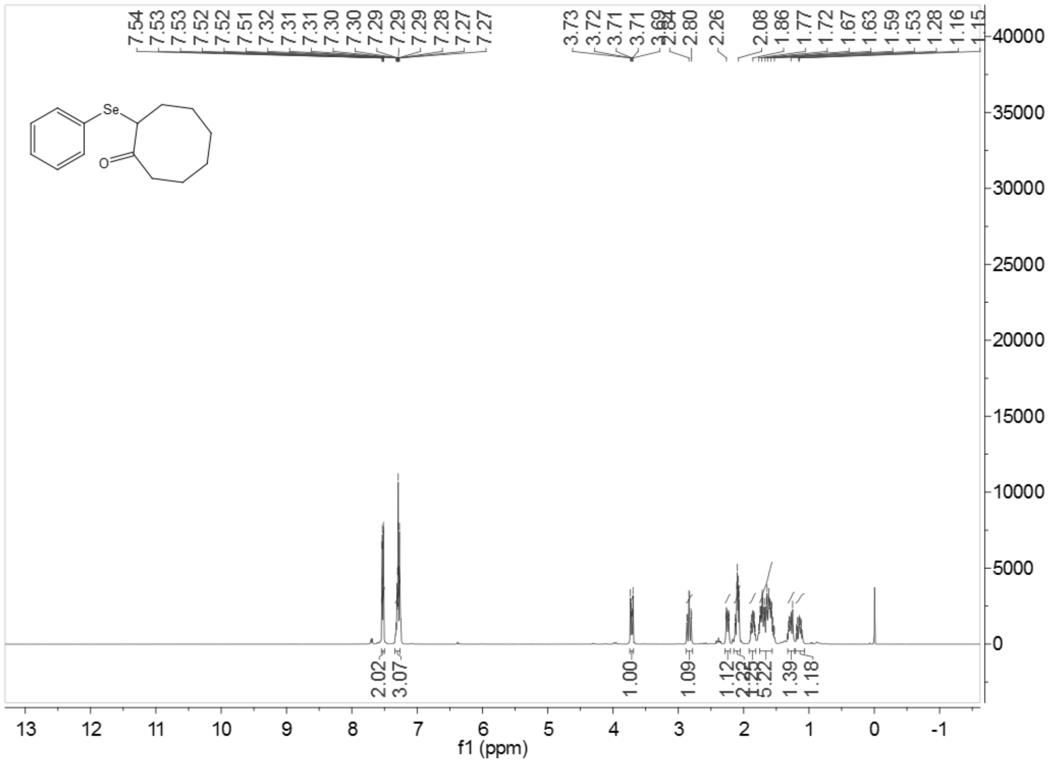

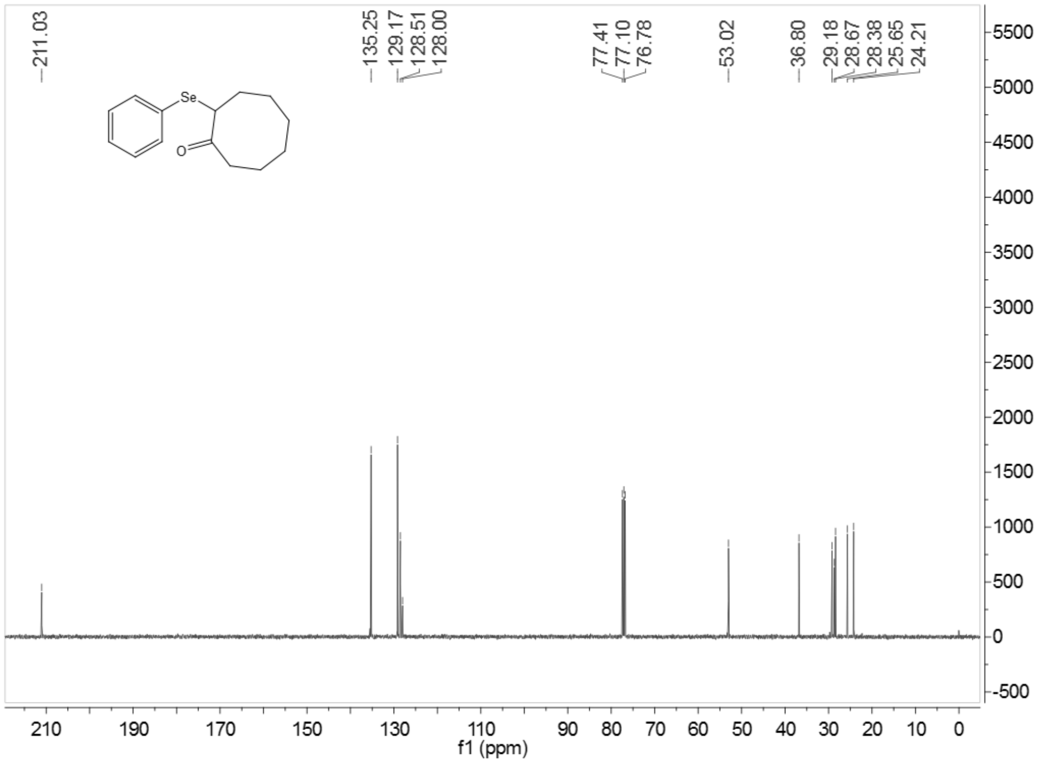

Figure S7. ^{1}H NMR and ^{13}C NMR of **3g**

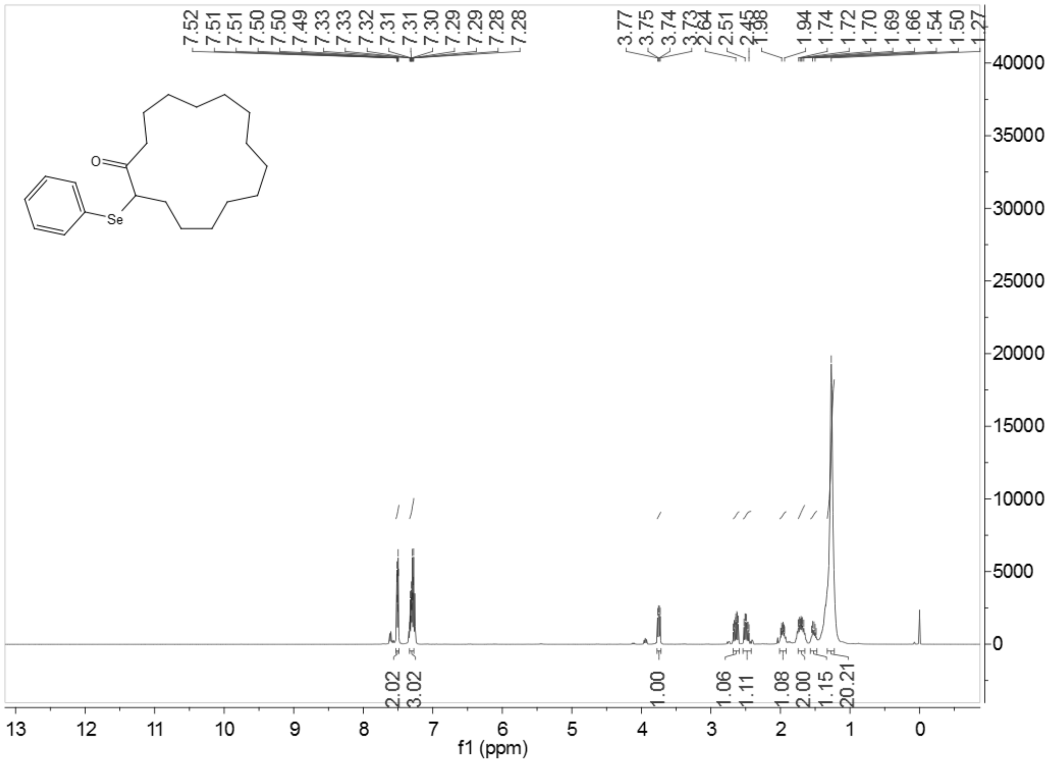

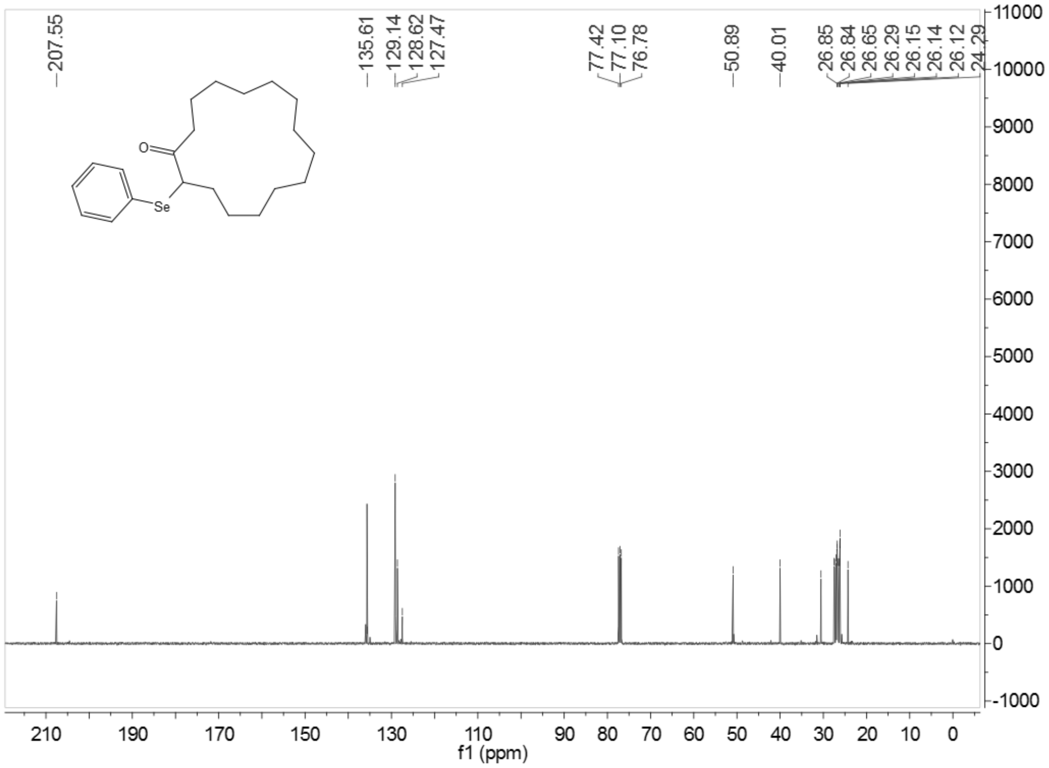

Figure S8. ^{1}H NMR and ^{13}C NMR of **3h**

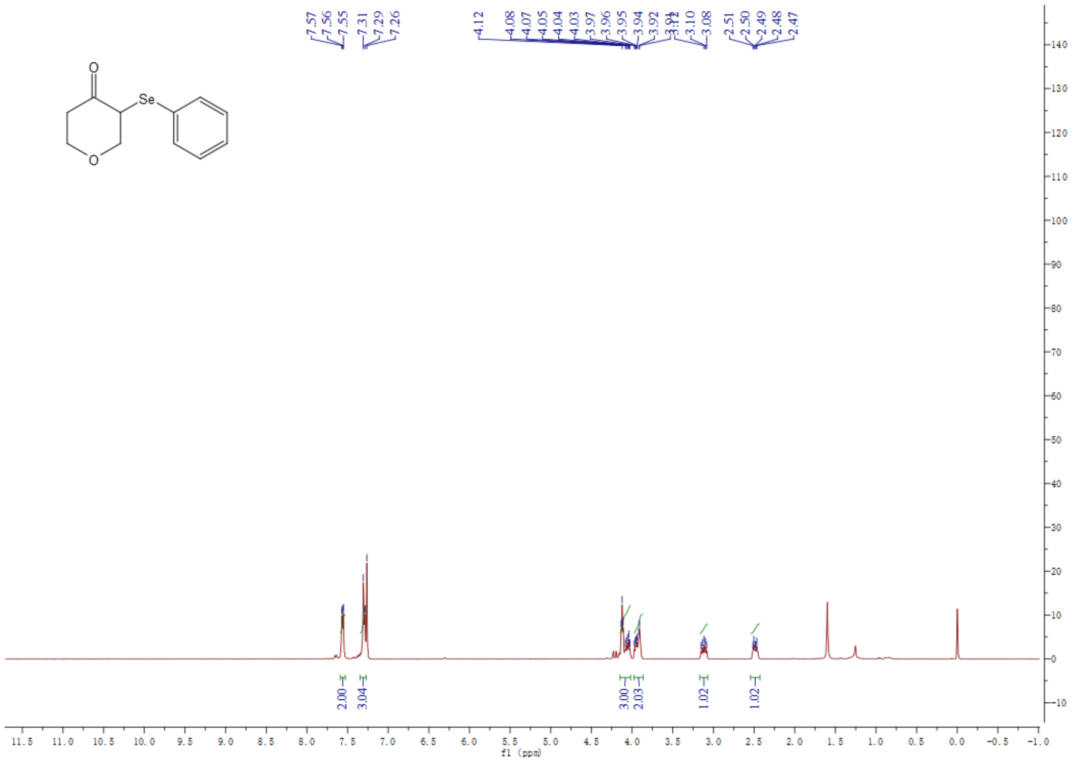

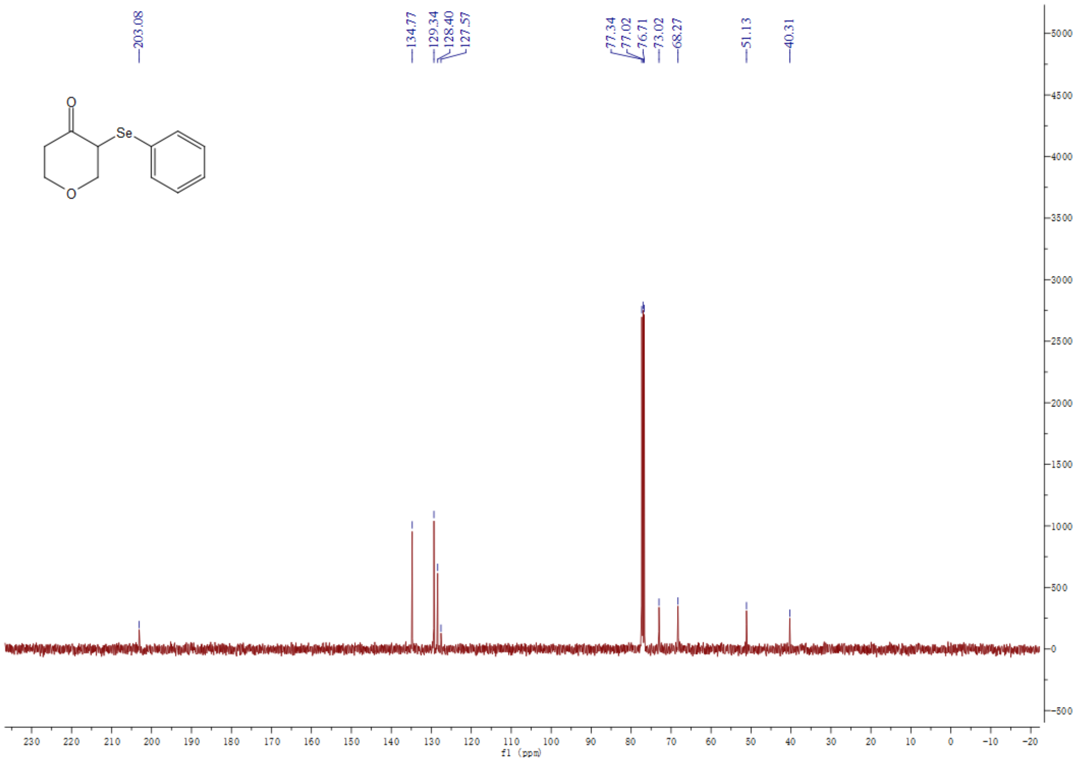

Figure S9. ^{1}H NMR and ^{13}C NMR of **3i**

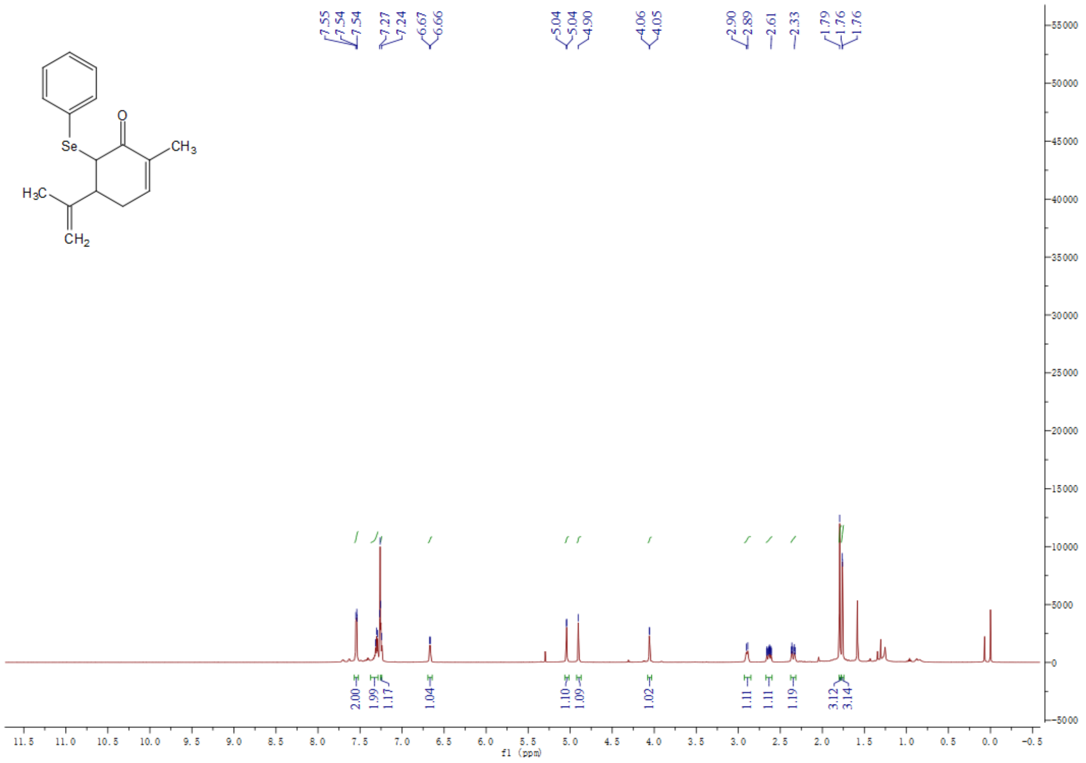

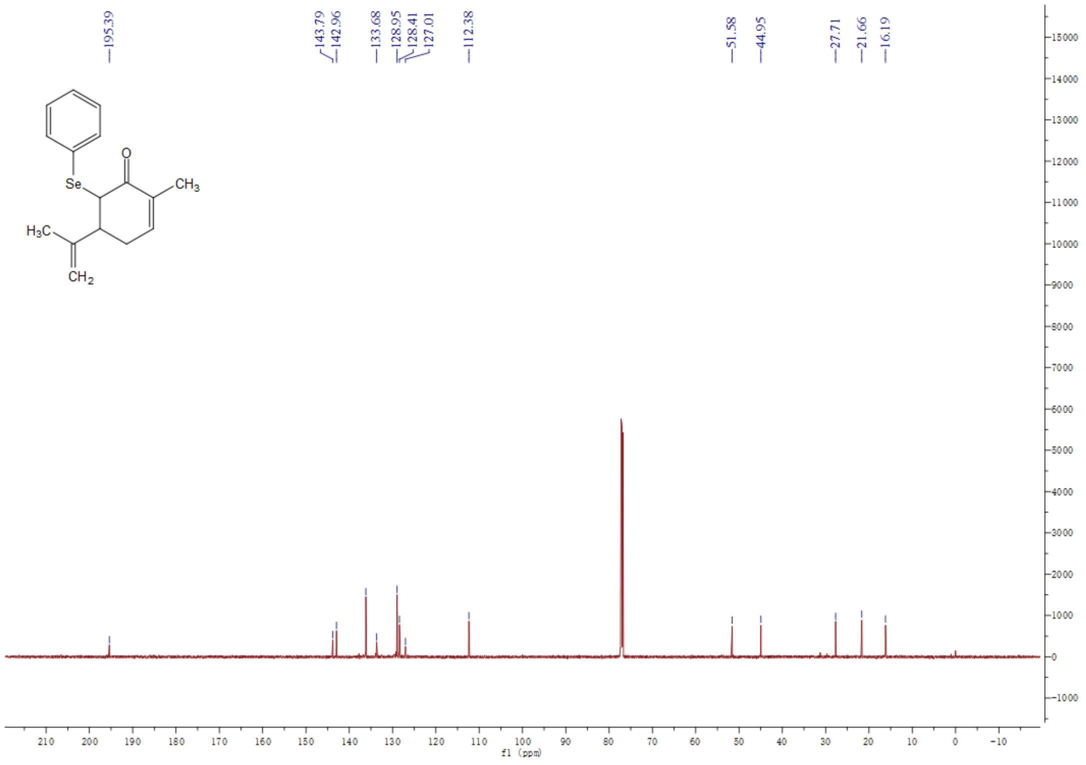

Figure S10. ^{1}H NMR and ^{13}C NMR of **3j**

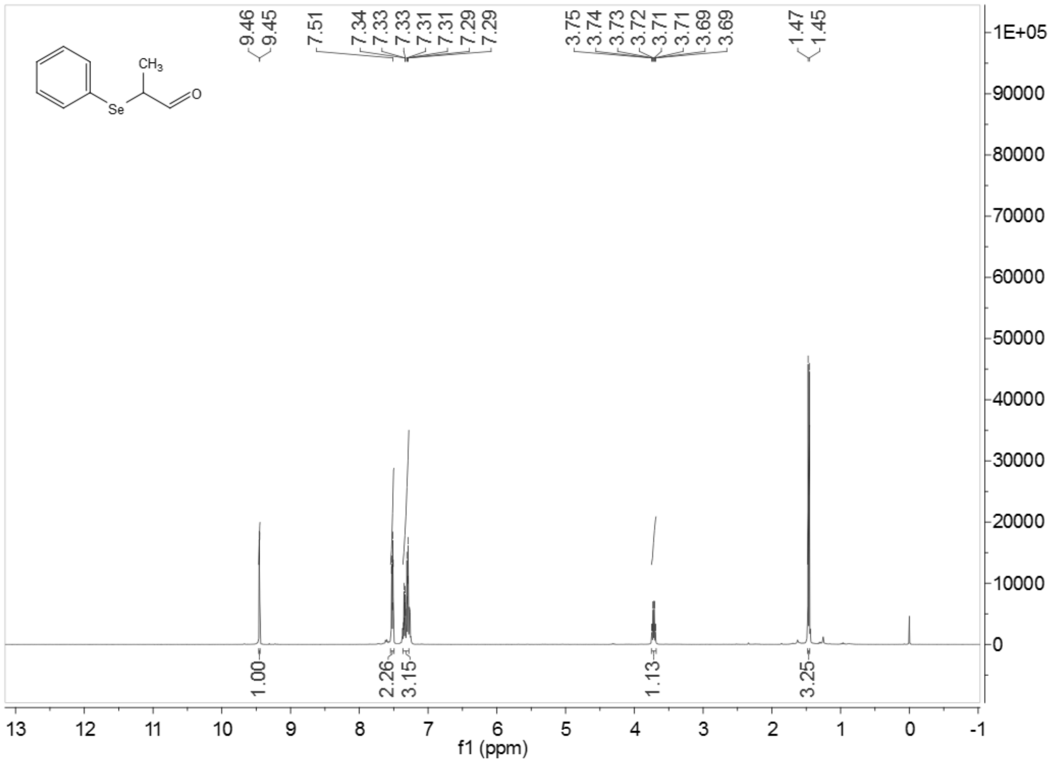

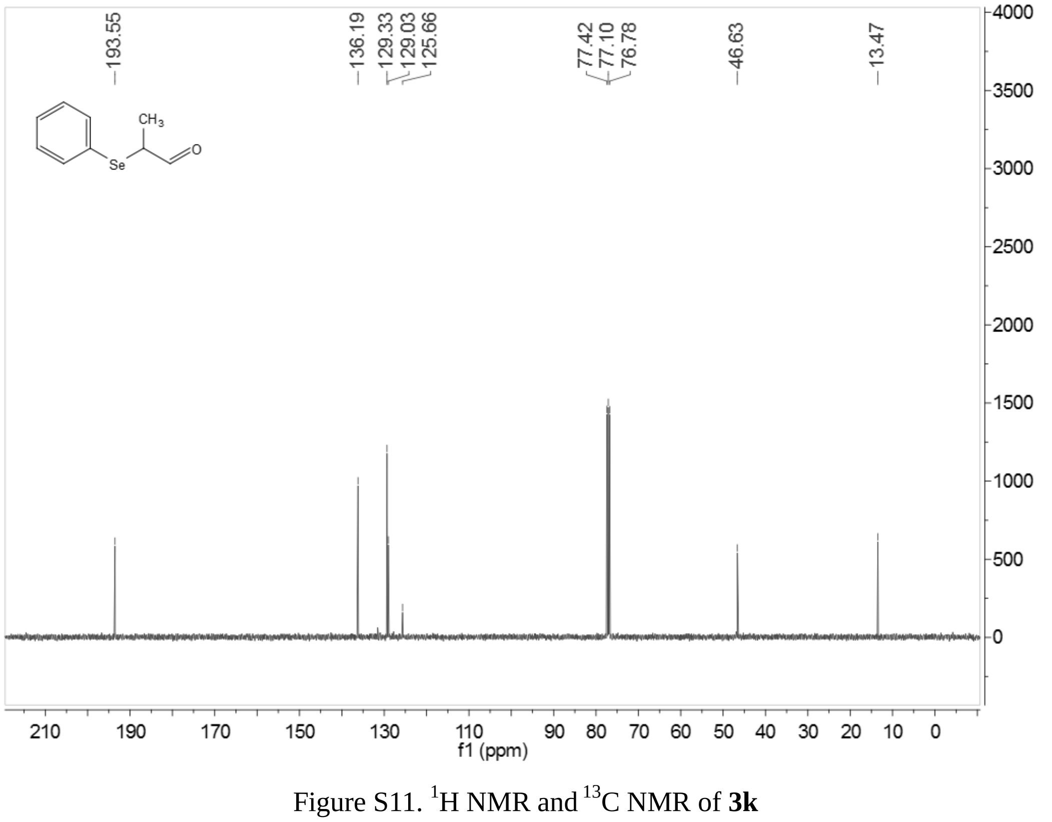

Figure S11. ^{1}H NMR and ^{13}C NMR of **3k**

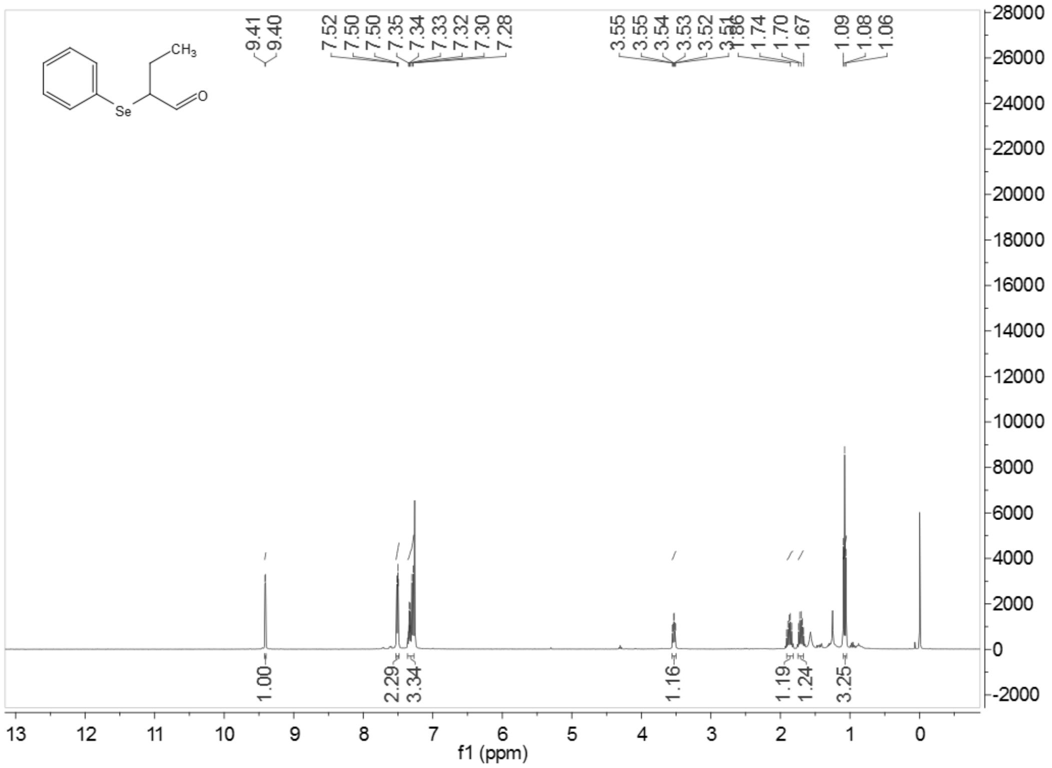

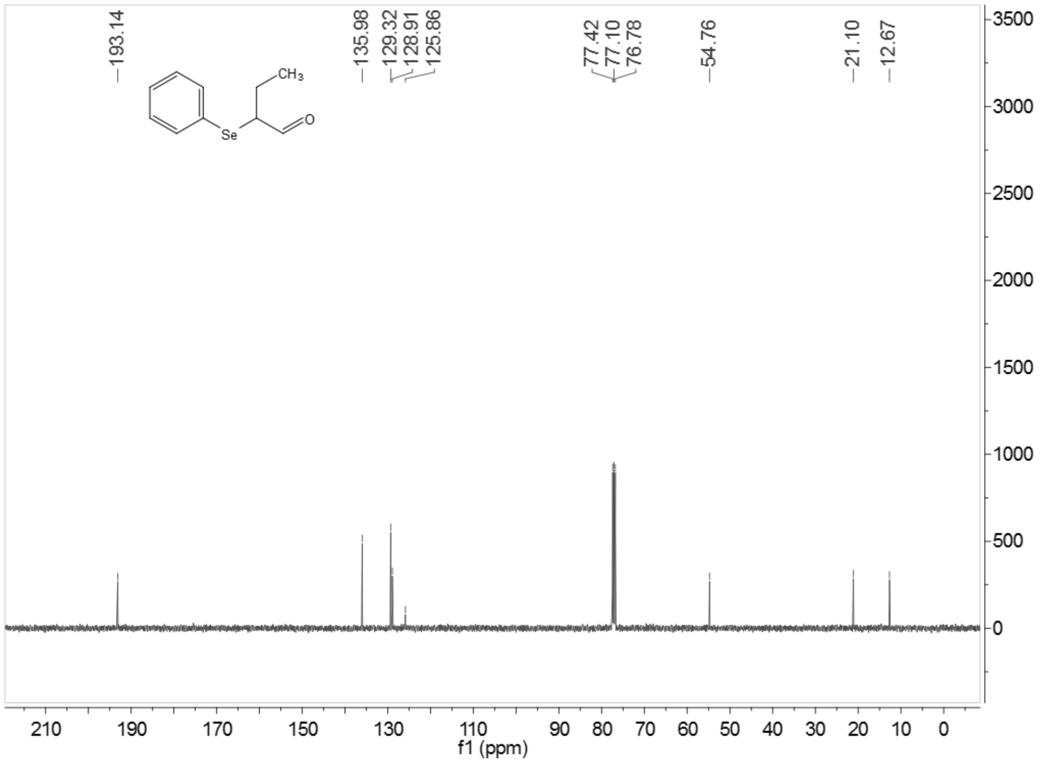

Figure S12. ^{1}H NMR and ^{13}C NMR of **3l**

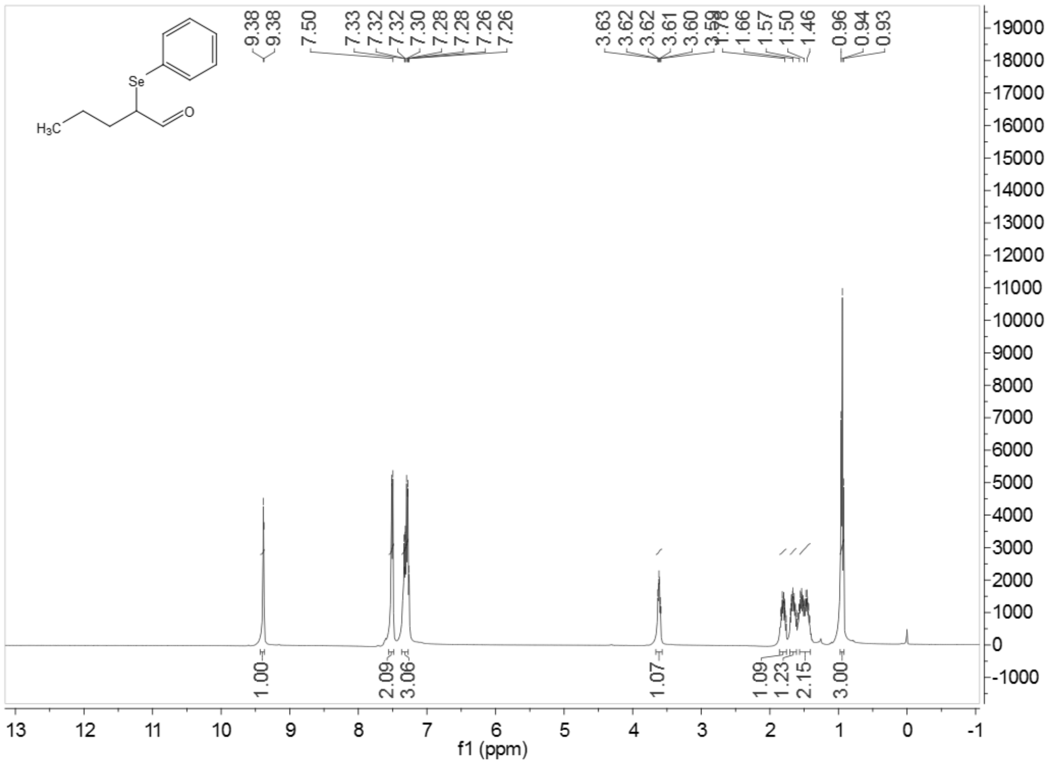

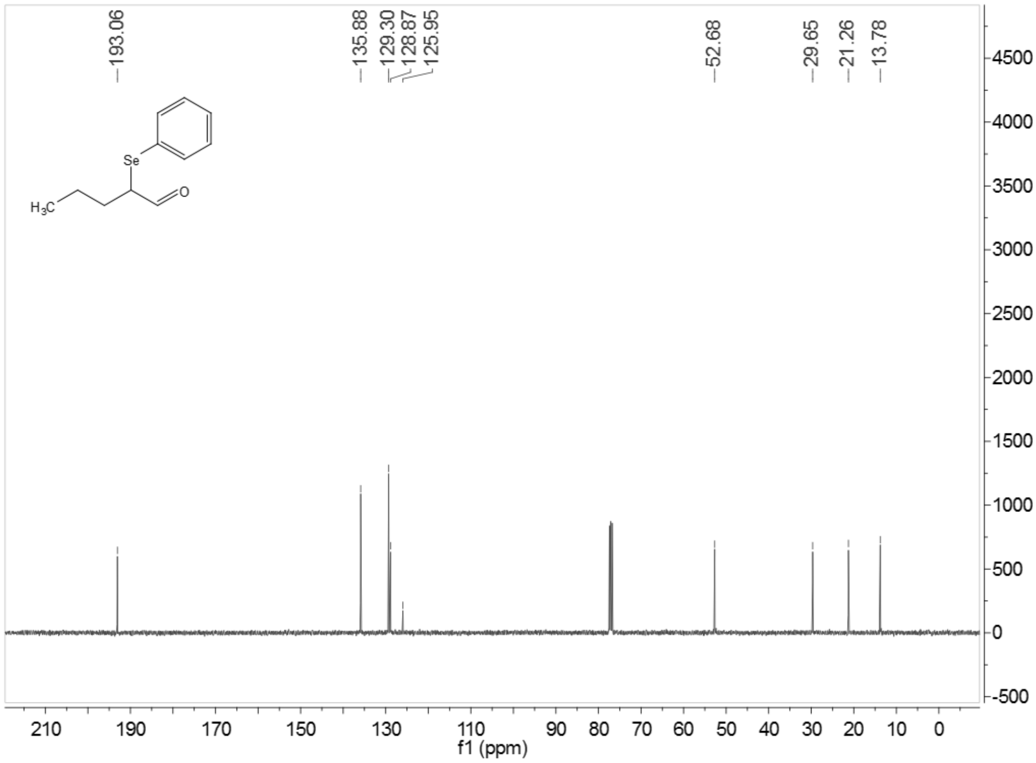

Figure S13. ^{1}H NMR and ^{13}C NMR of **3m**

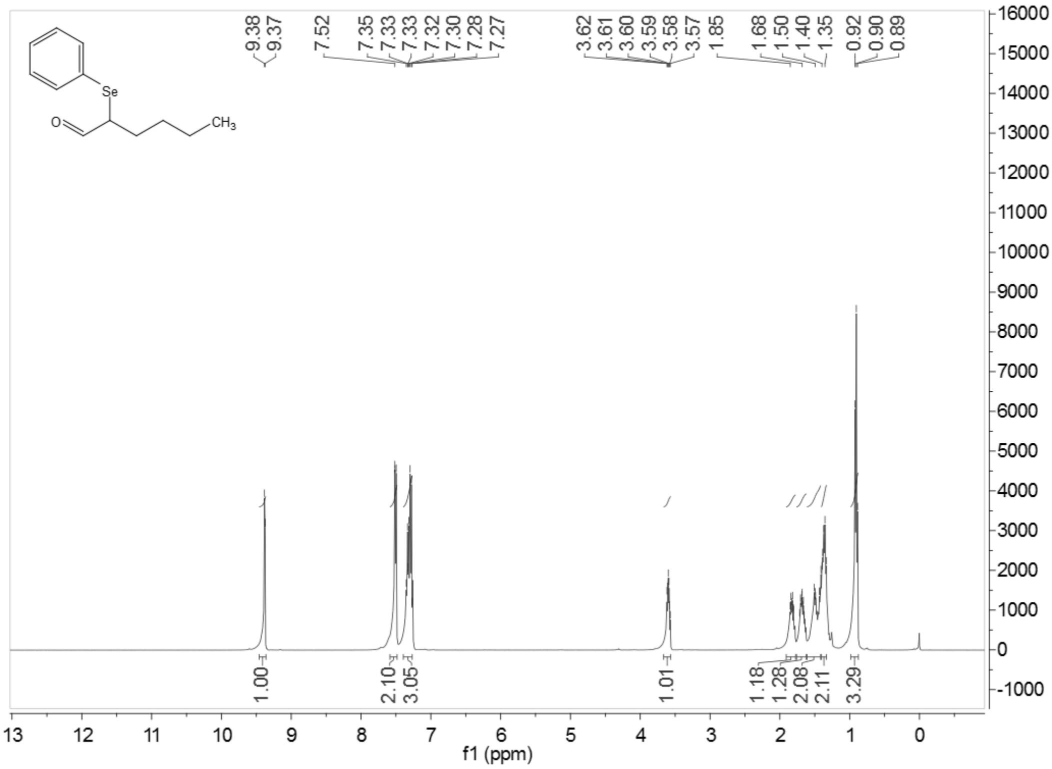

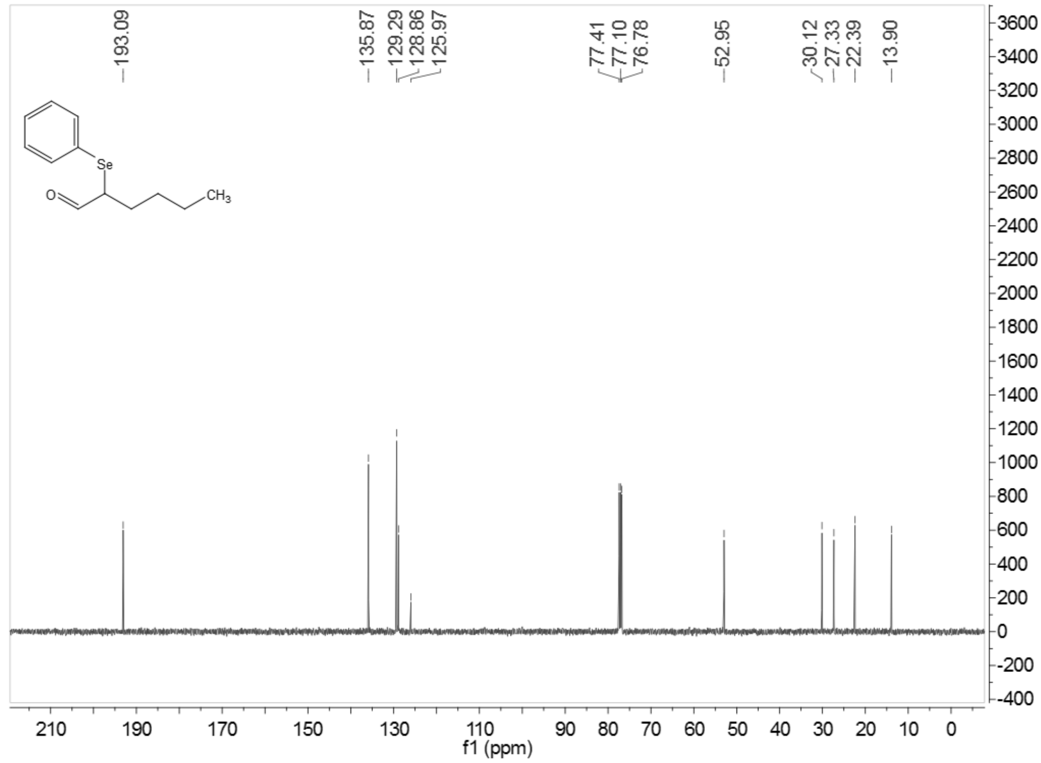

Figure S14. ^{1}H NMR and ^{13}C NMR of **3n**

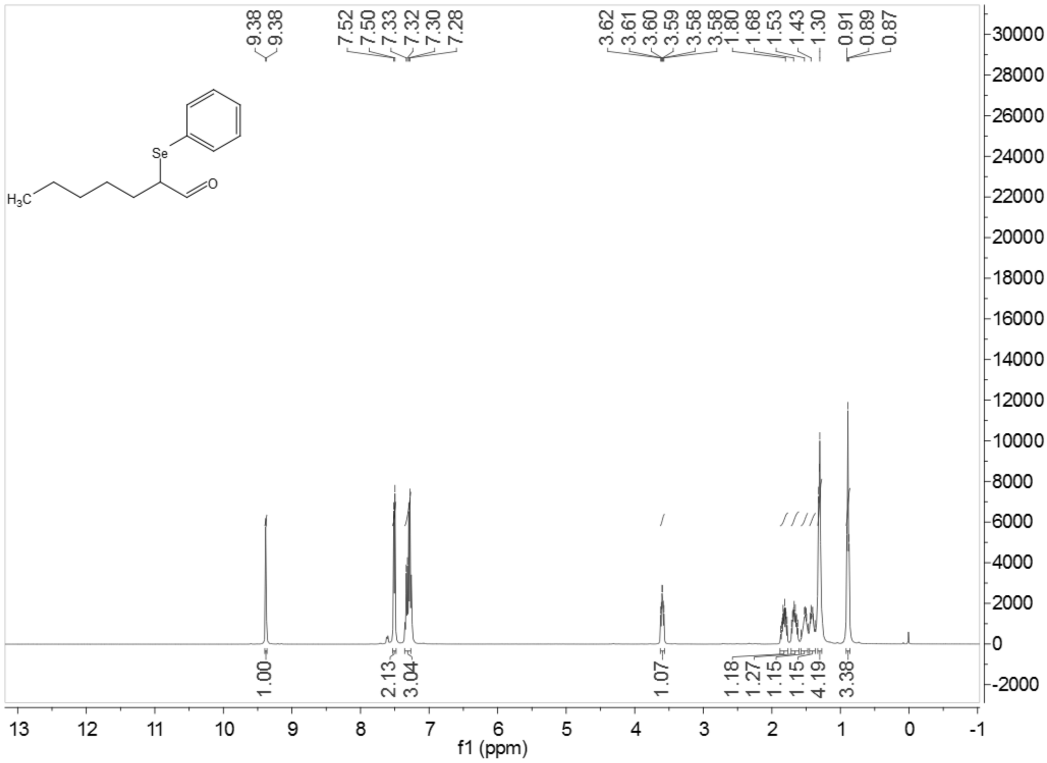

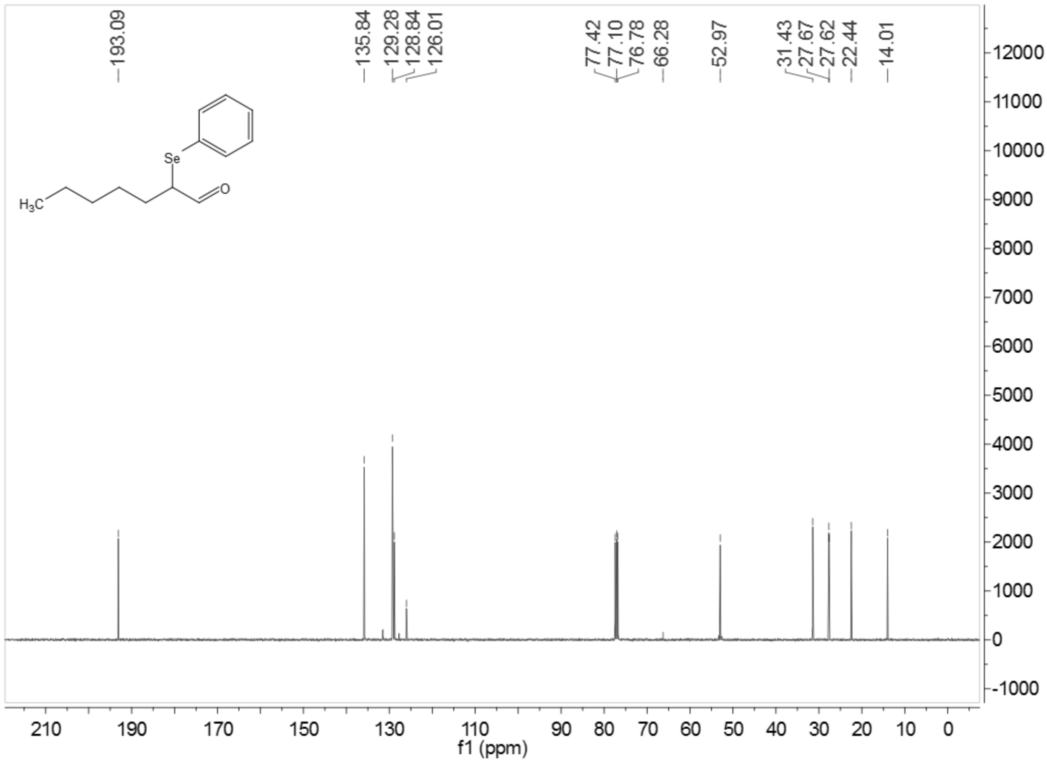

Figure S15. ^{1}H NMR and ^{13}C NMR of **3o**

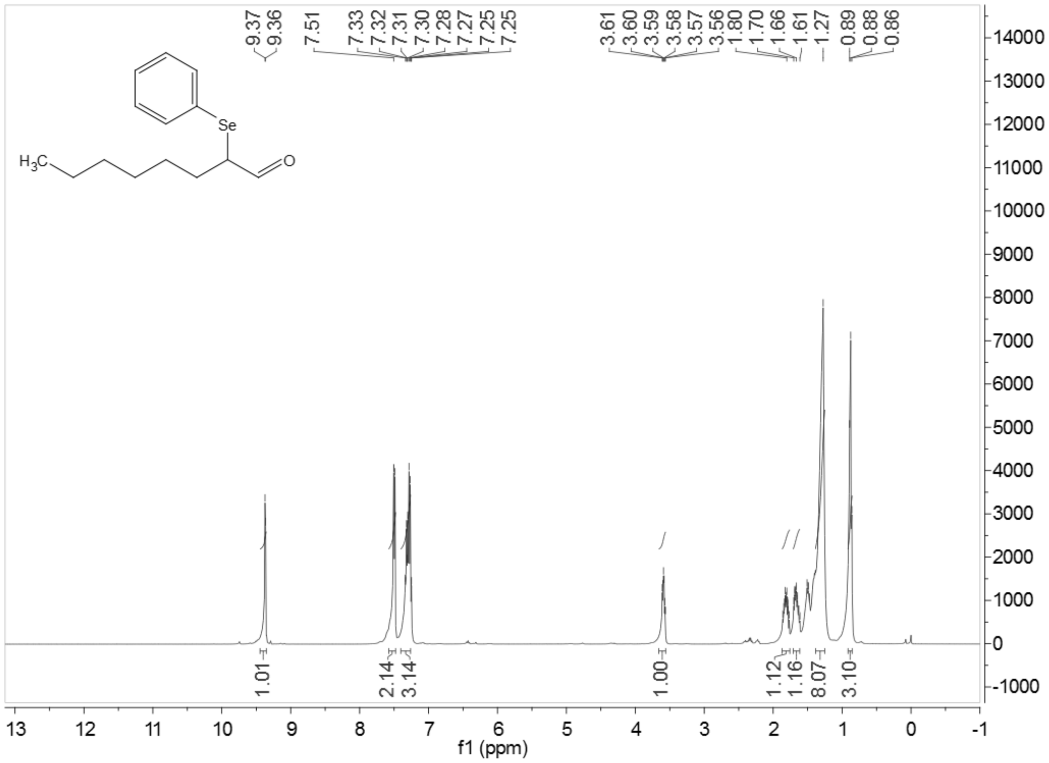

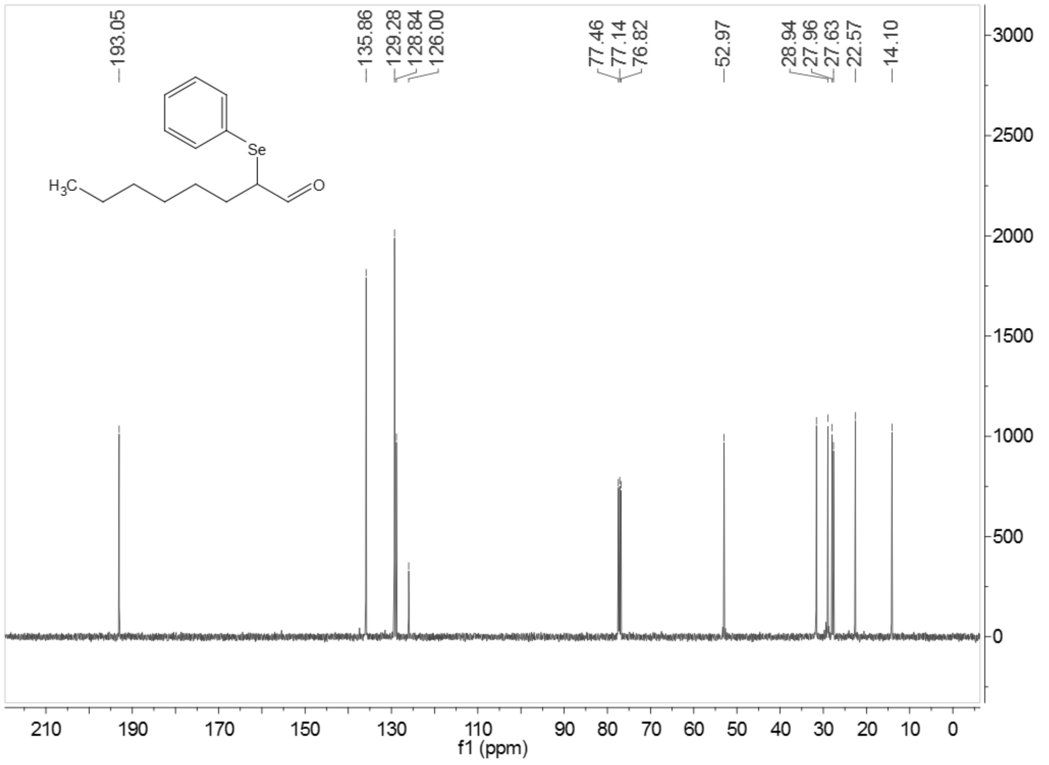

Figure S16. ^{1}H NMR and ^{13}C NMR of **3p**

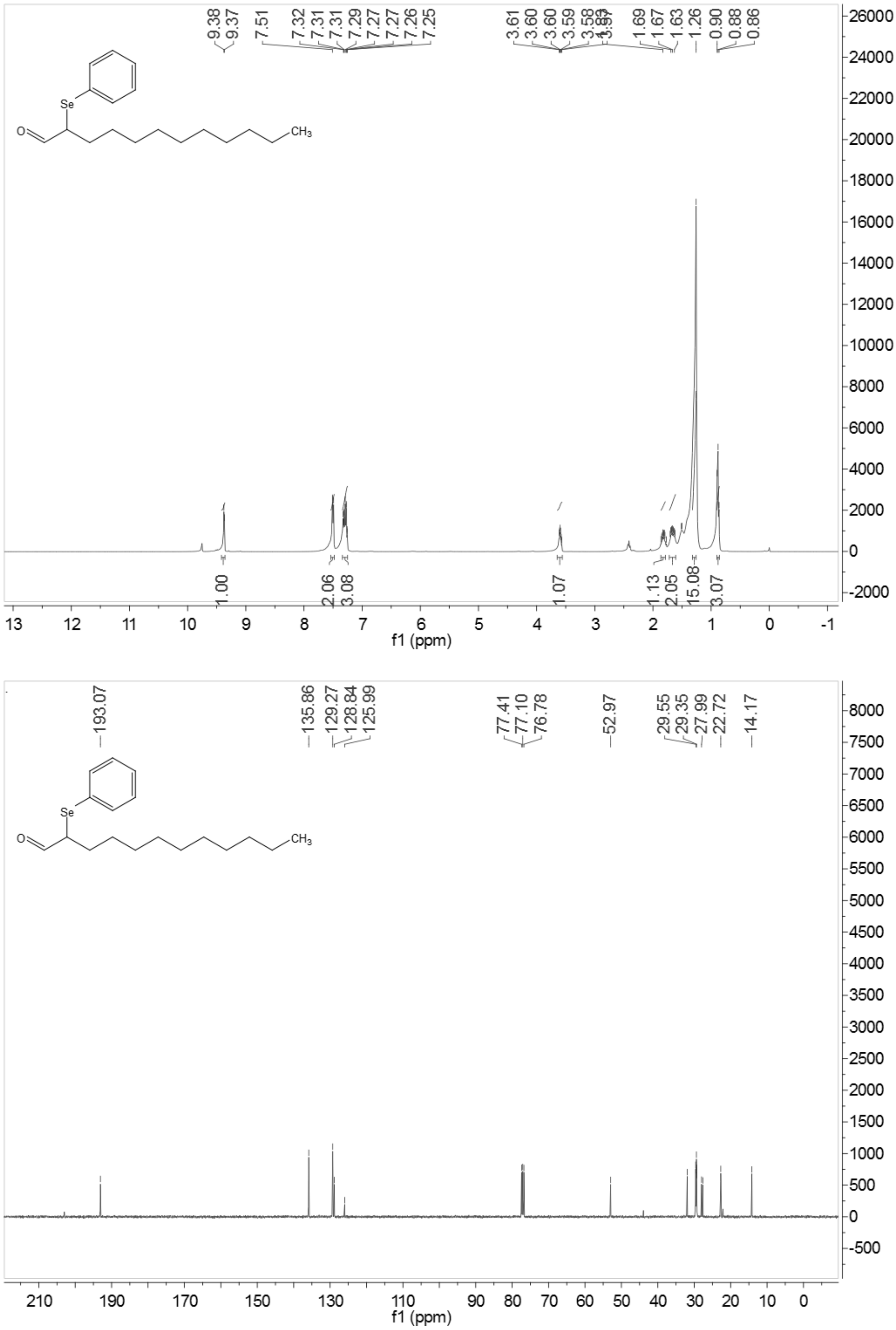

Figure S17. ^{1}H NMR and ^{13}C NMR of **3q**

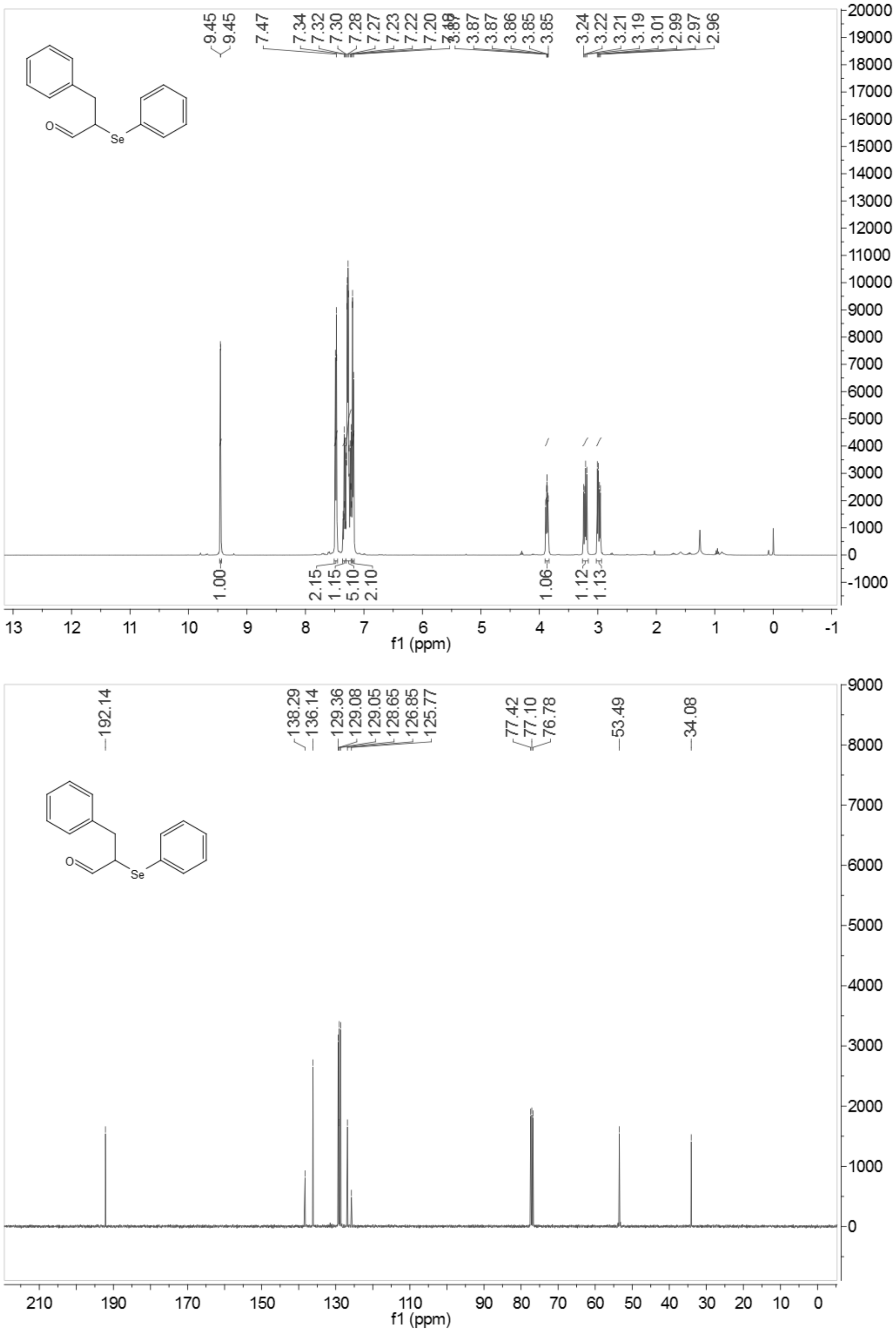

Figure S18. ^{1}H NMR and ^{13}C NMR of **3r**

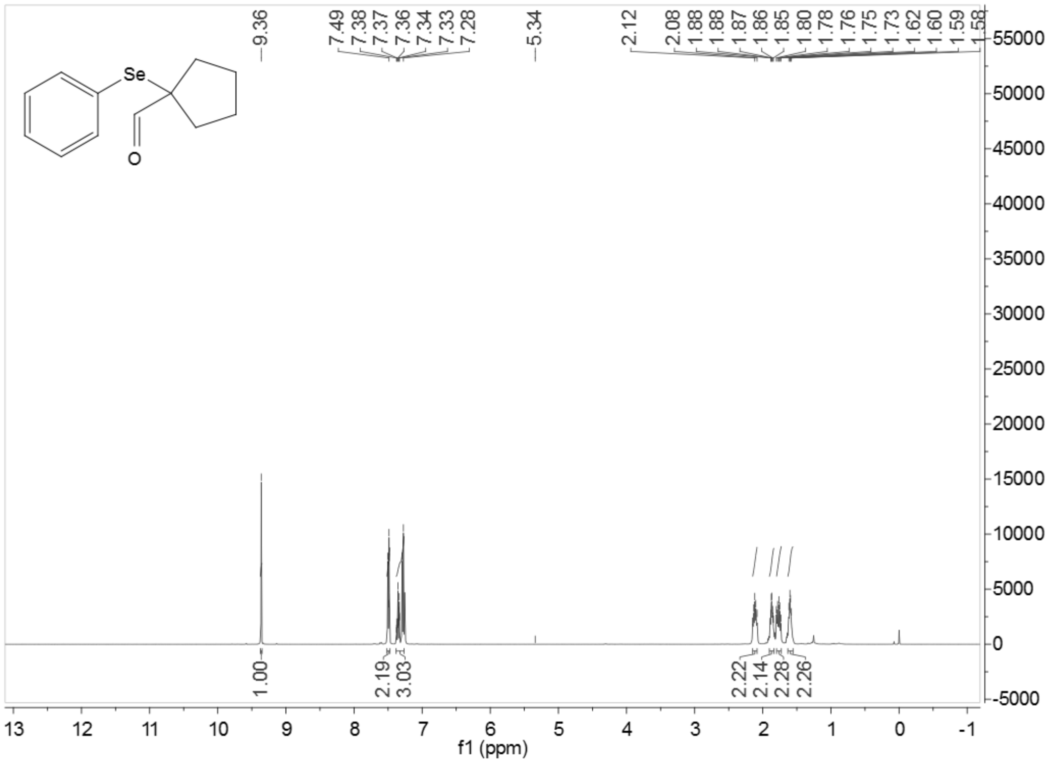

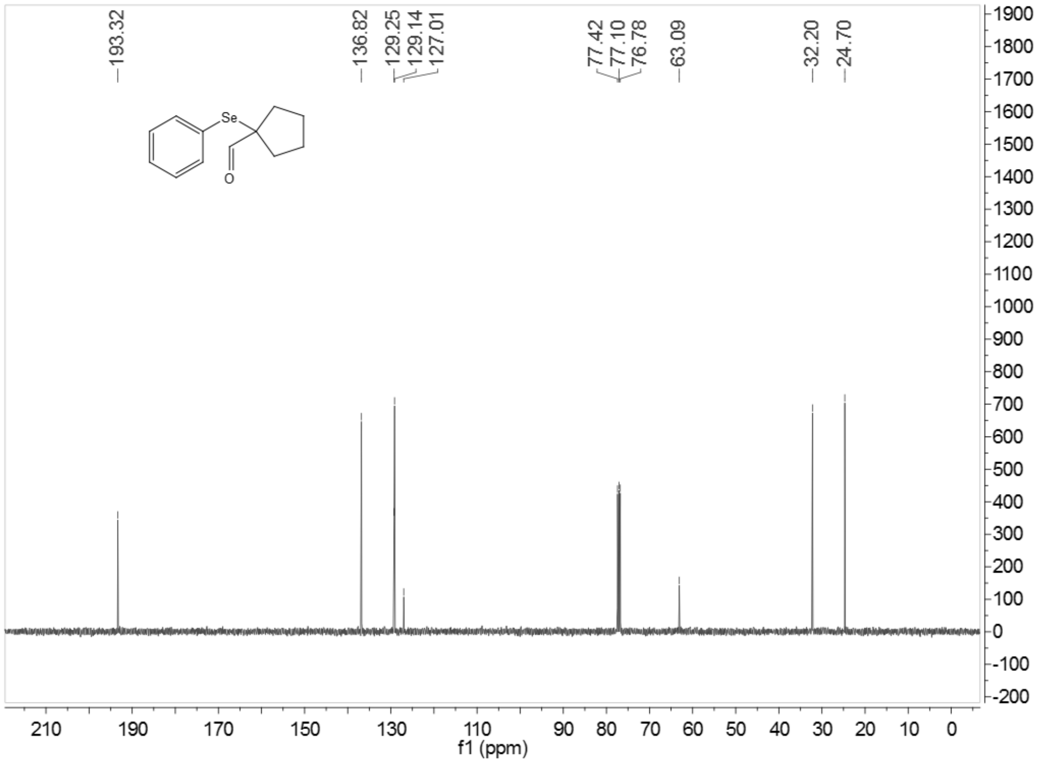

Figure S19. ^{1}H NMR and ^{13}C NMR of **3s**

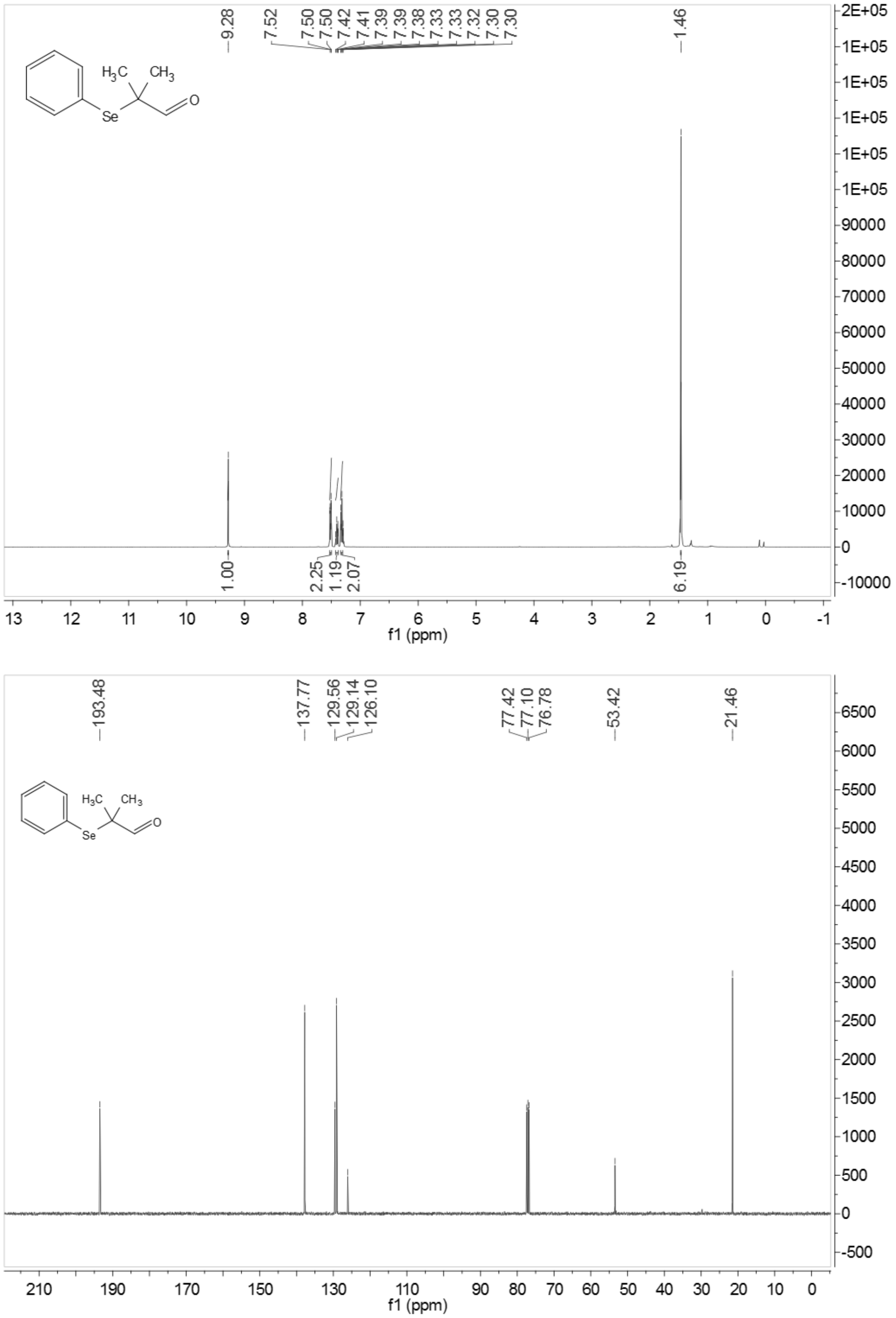

Figure S20. ^{1}H NMR and ^{13}C NMR of **3t**

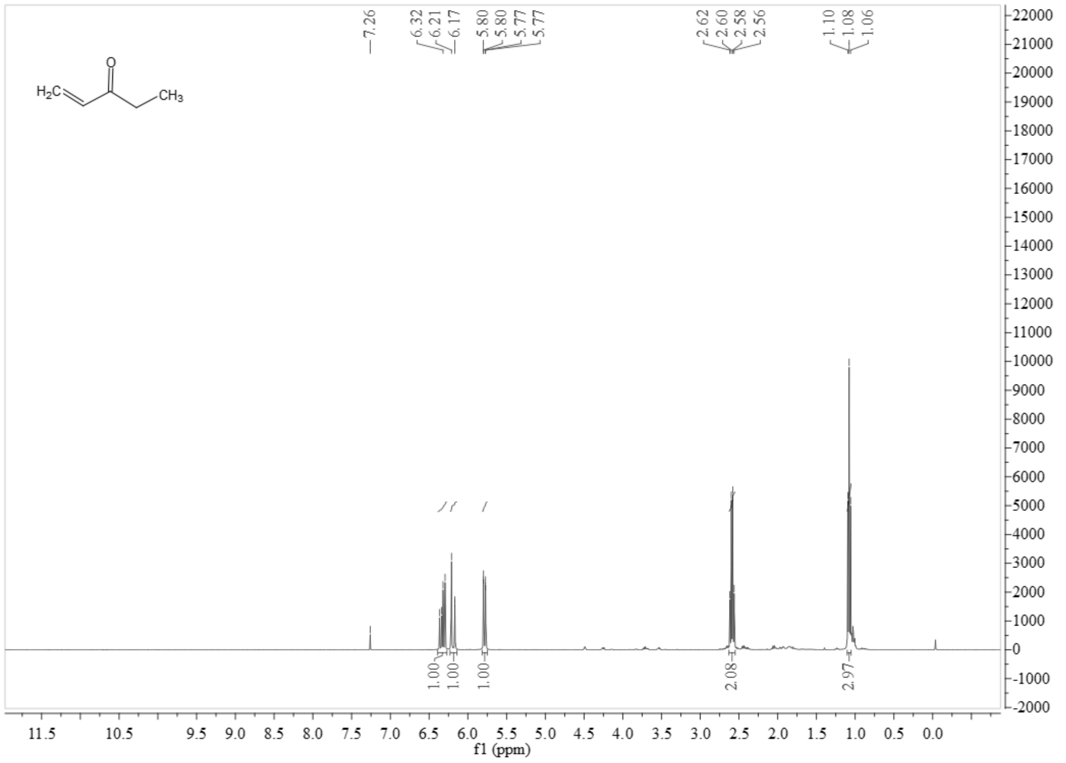

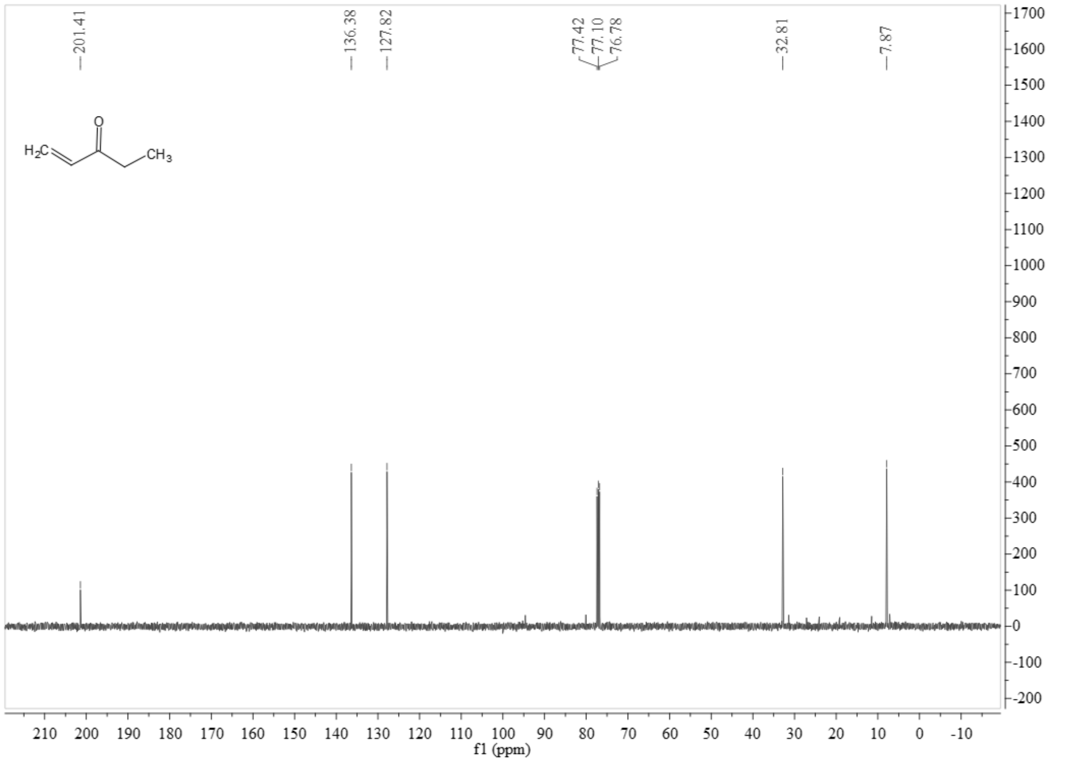

Figure S21. ^{1}H NMR and ^{13}C NMR of **4b**

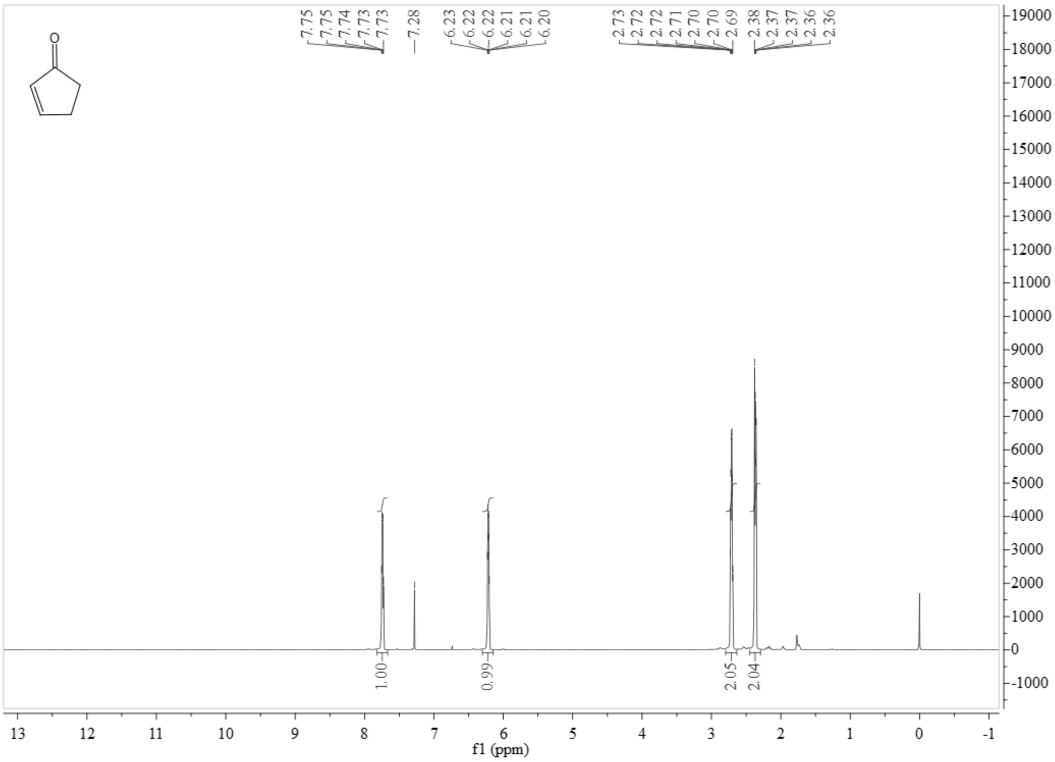

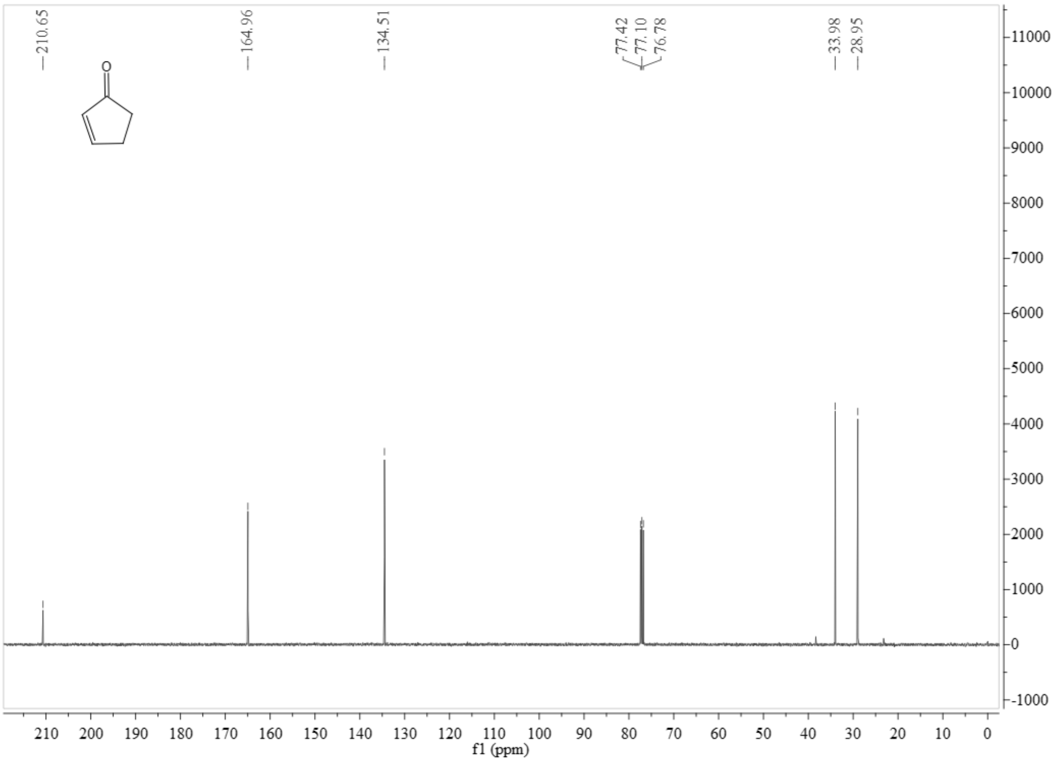

Figure S22. ^{1}H NMR and ^{13}C NMR of **4d**

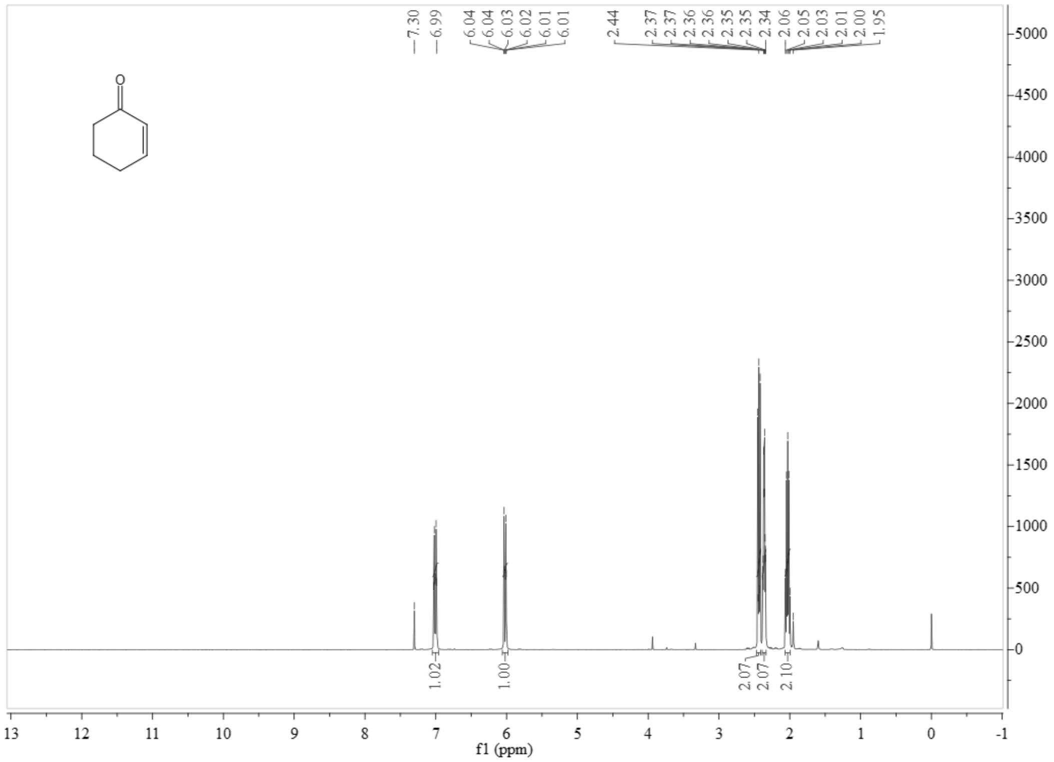

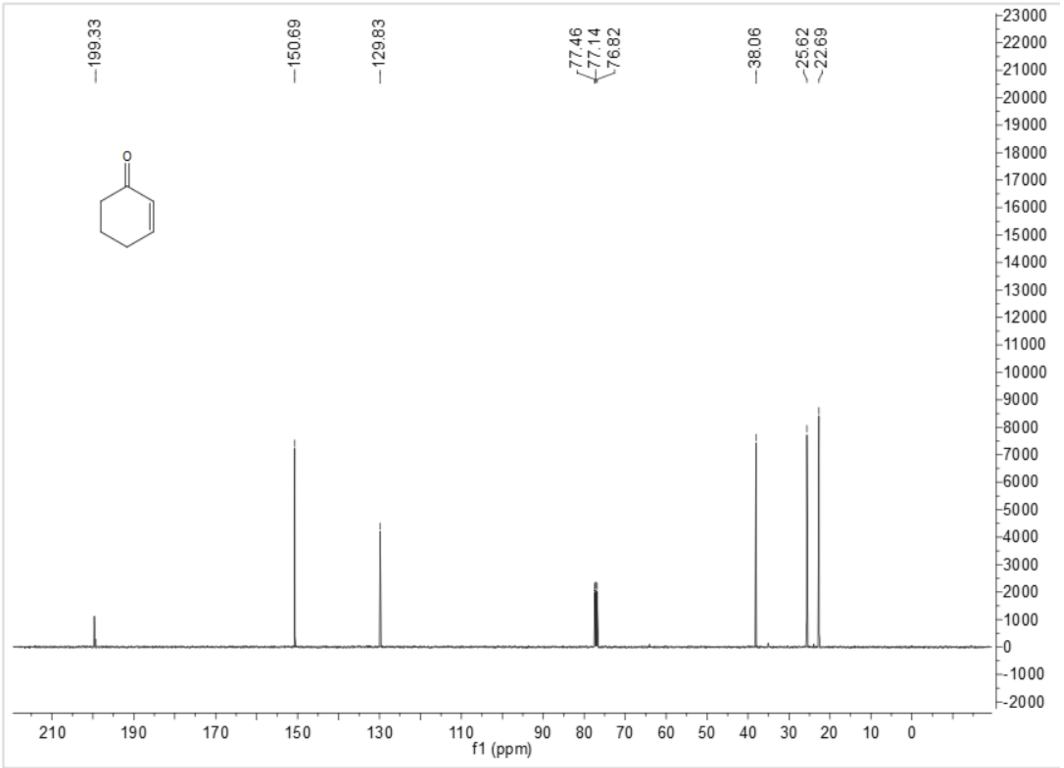

Figure S23. ^{1}H NMR and ^{13}C NMR of **4e**
